# Effects of age and sex on outcomes of the Q-Motor speeded finger tapping and grasping and lifting tests-findings from the population-based BiDirect Study

**DOI:** 10.3389/fneur.2022.965031

**Published:** 2022-09-30

**Authors:** Henning Teismann, Robin Schubert, Ralf Reilmann, Klaus Berger

**Affiliations:** ^1^Institute of Epidemiology and Social Medicine, University of Münster, Münster, Germany; ^2^George-Huntington-Institute, Münster, Germany; ^3^Institute of Clinical Radiology, University of Münster, Münster, Germany; ^4^Department of Neurodegenerative Diseases and Hertie-Institute for Clinical Brain Research, University of Tübingen, Tübingen, Germany

**Keywords:** manual motor performance, quantitative motor (Q-Motor), finger tapping, observational, cross-sectional, Purdue Pegboard, community-dwelling adults

## Abstract

**Background:**

Q-Motor is a suite of motor tests originally designed to assess motor symptoms in Huntington's disease. Among others, Q-Motor encompasses a finger tapping task and a grasping and lifting task. To date, there are no systematic investigations regarding effects of variables which may affect the performance in specific Q-Motor tests *per se*, and normative Q-Motor data based on a large population-based sample are not yet available.

**Objective:**

We investigated effects of age and sex on five selected Q-Motor outcomes representing the two core Q-Motor tasks speeded finger tapping and grasping and lifting in a community sample of middle-aged to elderly adults. Furthermore, we explored effects of the potentially mediating variables educational attainment, alcohol consumption, smoking status, and depressive symptoms. Moreover, we explored inter-examiner variability. Finally, we compared the findings to findings for the Purdue Pegboard test.

**Methods:**

Based on a sample of 726 community-dwelling adults and using multiple (Gaussian) regression analysis, we modeled the motor outcomes using age, sex, years in full-time education, depressive symptoms in the past seven days, alcohol consumption in the past seven days, and smoking status as explanatory variables.

**Results:**

With regard to the Q-Motor tests, we found that more advanced age was associated with reduced tapping speed, male sex was associated with increased tapping speed and less irregularity, female sex was associated with less involuntary movement, more years of education were associated with increased tapping speed and less involuntary movement, never smoking was associated with less involuntary movement compared to current smoking, and more alcohol consumed was associated with more involuntary movement.

**Conclusion:**

The present results show specific effects of age and sex on Q-Motor finger tapping and grasping and lifting performance. In addition, besides effects of education, there also were specific effects of smoking status and alcohol consumption. Importantly, the present study provides normative Q-Motor data based on a large population-based sample. Overall, the results are in favor of the feasibility and validity of Q-Motor finger tapping and grasping and lifting for large observational studies. Due to their low task-complexity and lack of placebo effects, Q-Motor tests may generate additional value in particular with regard to clinical conditions such as Huntington's or Parkinson's disease.

## Introduction

Motor performance of the hands is reflected in strength, speed, dexterity, and hand preference. Deficits in motor performance are found in many different (e.g., neurological) conditions. A core set of functional measures, among them finger tapping speed, grip strength, and Grooved and Purdue Pegboard (dexterity, speed, bimanual coordination) has proven useful for identifying manual motor impairment (1).

Q-(“Quantitative”-)Motor is a suite of objective, non-invasive, and easy-to-administer motor tests originally designed to assess motor symptoms in biomarker studies and clinical trials targeting Huntington's disease (HD). Q-Motor was recently developed to provide an alternative to the “Unified Huntington's Disease Rating Scale Total Motor Score” (UHDRS-TMS) to address concerns such as limited sensitivity for dysfunction and change, restriction to manifest stages of disease, intra- and inter-rater variability, subjective error, and rater-induced placebo-effects (2).

The Q-Motor apparatus encompasses pre-calibrated, temperature-independent force (-torque) transducers and 3D position-orientation sensors, enabling standardized measurements across time points and sites. Processing and (pre-) analysis of Q-Motor data can be carried out in a blinded and automated manner (2). The core Q-Motor suite includes six different tests. Two of these tests, which are routinely applied in HD research, are speeded finger tapping (digito-motography) (3, 4) and grasping and lifting (manu-/ choreo-motography) (4–6).

The Q-Motor speeded finger tapping test is one specific variant of the classical and widely used neuropsychological finger tapping test [for overviews, see (1, 7, 8)]. The latter was originally designed to measure self-directed manual motor speed and fine motor control, though performance also depends on kinesthetic and visual abilities as well as motivational status. The finger tapping test has been used with regard to and has proven sensitive to a wide variety of conditions, among them e.g., chronic alcoholism, Korsakoff's syndrome, closed-head injury, chronic pain, Multiple Sclerosis, Parkinson's disease (PD), and HD. While the task itself may appear simple, it actually is a complicated motor sequencing and motor inhibition-excitation task (9, 10). The purpose of the test is to assess subtle motor (and other cognitive) impairments. Noteworthy, interpretation and comparison of test results is complicated by the use of different tapping devices [manual vs. electronic/computerized apparatus vs. smartphone app; e.g., Halstead-Reitan Finger Tapping Test (11), Computerized Finger Tapping Test (12), T3 computer-assisted finger tapping task (13), WPS Electronic Tapping Test (12), CNS Vital Signs (14), potentiometer (15), or Android mobile application (16)] as well as different administration techniques, e.g., with regard to the number of trials or trial duration, both of which may result in subtle performance differences. In the past, mainly the manual apparatus has been used, and tapping speed was operationalized as the mean number of taps across five 10 s trials for which the total number of taps was within a range of five. More recently, further measures were applied, e.g., inter-tap interval (17), inter-tap variability (18), tap initiation time (19), tap down-time (20), or the occurrence of abnormal finger movements (21). All these core measures can be assessed with the Q-Motor digito-motography tapping test (3).

The Q-Motor grasping and lifting test was specifically designed to apply object grasping, lifting, holding, and transport tasks in order to investigate e.g., coordination of prehensile forces during precision grip (22), movement trajectories and force control during object transport (23), and quantity of involuntary movements, e.g., chorea in HD (5, 24) or L-Dopa induced dyskinesia in PD (25). The use of electro-magnetic position-angle sensors thereby enables standardized tracking of object position and orientation in all three planes.

Q-Motor tests and measures have been used in a number of multi-center trials and biomarker studies, among them e.g., TRACK-HD (26), TRACK-ON-HD (27), and PRIDE-HD (28) [see (2) for further trials], which investigate HD course, symptomatic, or disease-modifying treatments. However, what is currently lacking and may be important for the design of future studies employing Q-Motor measures are (i) systematic investigations into variables which may influence the performance in specific Q-Motor tests *per se*, and (ii) normative data from large population-based samples. For instance, due to lack of normative data, Q-Motor findings in clinical samples are currently sometimes hard to interpret.

Many previous investigations have consistently demonstrated effects of age and sex on finger tapping performance [for overviews, see (1, 7, 8)]. For grasping and lifting performance, such investigations are still pending. With regard to age, previous studies found increased tapping speed with increasing age in children [e.g., (29, 30)] and usually decreased speed [e.g., (9, 17, 31–38)] and increased variability (35) with advancing age in adults, particularly from the fifth decade on, but also starting as early as the third decade (35). With regard to sex, previous studies found increased tapping speed in boys compared to girls (29) and usually in males compared to females [e.g., (9, 11, 12, 18, 33, 35, 39–42)]. Moreover, there are conflicting results indicating that males may either show lower (18) or higher (20) inter-tap variability compared to females. Whether observed sex effects were influenced by body or hand size differences between the sexes remains currently unresolved.

A recent study (30) investigated effects of age and sex on Q-Motor outcomes in a community-dwelling sample (*N* = 29) of children and adolescents aged 6 to 17 years. The study included measures of speeded finger tapping and grasping and lifting. The results indicated robust age-related trends in both speeded finger tapping and grasping and lifting performance. Regarding finger tapping, for instance there were associations of advanced age with decreased (i.e., better) tap inter-onset interval mean and standard deviation, and with increased (i.e., better) tapping frequency revealing improvements in motor coordination with development. With respect to grasping and lifting, there were e.g., associations of advanced age with decreased (i.e., better) orientation and (particularly) position indexes, synonymous of less motor impersistency or fluctuation, which also supports maturation of motor development and shows that these effects were detectable with sensitive technology in a rather small sample size.

In addition to age and sex, there are other variables which may influence motor performance (and which, at the same time, are influenced by the age and the sex of subjects). Such variables could act as mediators in the analysis of effects of age and sex on motor performance. For instance, effects of alcohol on motor control [e.g., delay of reaction times, adverse effects on cognitive and motor processing, or the transient alleviation of neurological symptoms (e.g., essential tremor)] are well-recognized, and withdrawal of alcohol may trigger movement disorders (43). Moreover, smoking behavior (or nicotine, respectively) may affect motor performance. For instance, acute effects of nicotine and smoking on finger tapping performance have been reported previously [e.g., (44–47)]. Furthermore, depression is known to typically affect psychomotor skills, with either agitation or retardation considered core symptoms [cf. (48), for a review].

The primary objective of the present study was to investigate effects of age and sex on five selected Q-Motor outcomes representing the two core Q-Motor tasks speeded finger tapping and grasping and lifting in a community sample of middle-aged to elderly adults. To learn more about potential effects of age and sex on Q-Motor performance is crucial to improve the interpretation of existing Q-Motor data from clinical samples. Furthermore, the present study explored effects of the potentially mediating variables educational attainment, alcohol consumption, smoking status, and depressive symptoms. Moreover, given that the tasks were explained and supervised by a number of different study nurses, we explored inter-examiner variability to get an impression regarding the robustness of the test results with respect to the executing personnel. Additionally, the present study compared the findings for the Q-Motor outcomes to findings for the Purdue Pegboard test [for overviews, see (1, 7)], a classical neuropsychological test to assess hand and finger dexterity. The Purdue Pegboard is a well-established test that allows to explore the link between Q-Motor measures with different features of motor coordination. Finally, this study provides normative Q-Motor data from a large population-based sample [*N* = 726; the largest dataset investigated so far included about 120 control subjects in the TRACK-HD study (26)], which is not yet available, and which can be used as a comparative data set in future research studies. For instance, the normative Q-Motor data will enable future studies to compare their data to this dataset and assess the behavior of Q-Motor measures in population-based studies and beyond. Moreover, the normative data will be helpful to inform the design of future research and interventional trials.

## Methods

### The BiDirect Study

The BiDirect Study (49, 50) is an observational, prospective cohort study originally designed to investigate the bidirectional relationship between depression and (subclinical) arteriosclerosis. BiDirect enrolled three cohorts of participants: (i) patients hospitalized due to an acute episode of depression at the time of recruitment, (ii) patients shortly after myocardial infarction or an acute coronary event at the time of recruitment, and (iii) population-based control subjects randomly invited from the registry of the city of Münster, Germany. The BiDirect Study was approved by the ethics committee of the University of Münster and the Westfalian Chamber of Physicians in Münster, North-Rhine-Westfalia, Germany. Written informed consent for participation was obtained from all participants.

### Sample

The subjects analyzed here were those members of the BiDirect Study control cohort who (i) participated in the second BiDirect visit (*N* = 800), and who (ii) contributed Q-Motor or Pegboard data at this visit (*N* = 763), and who (iii) had not to be excluded from Q-Motor or Pegboard data analysis based on plausibility checks of the data (*N* = 726). Reasons due to which participants were excluded encompassed acute injuries of fingers/ hands/ arms, missing fingers/ parts of fingers, disability, acampsia, hand deformation, rheumatism, diagnosis of Multiple Sclerosis, diagnosis of PD, essential tremor, and vision deficits. The sample size was further reduced in analyses of specific Q-Motor or Pegboard outcomes due to missing data in such outcomes or, if so, due to missing data in explanatory variables used during display or statistical modeling of motor outcomes.

### Motor testing and motor data processing in the BiDirect Study

Q-Motor testing was performed during the second and third out of in total four BiDirect examinations, which took place between 2010 and 2020. Purdue Pegboard testing was performed during all four BiDirect examinations.

Q-Motor was executed in a quiet room as the first unit of the BiDirect neuropsychological assessment module. The Purdue Pegboard test was conducted subsequently. In each case, the participants were instructed and assessed by trained and certified examiners based on written standard operation procedures. Participants were not allowed to consume caffeine in the 30 min time period prior to neuropsychological assessment onset.

The BiDirect Study focused on two core Q-Motor tests: (i) speeded finger tapping (digitomotography), and (ii) grasping and lifting (manumotography and choreomotography). Grasping and lifting was performed first, speeded finger tapping was performed subsequently. Both tasks were executed with each hand separately; for each task and hand, three trials were conducted. The signals were recorded by means of a customized software tool (WinSC, Umeå University, Sweden) installed on a personal computer running Windows XP.

Prior to Q-Motor testing, the height of the chair was adjusted such that the forearms were approximately parallel to the floor, with the elbows at ~90° when the tasks were performed.

Regarding Q-Motor grasping and lifting, the task was to grasp a grip device with affixed position sensor (Polhemus Fastrak, VT, USA) and force transducer (Mini-40, ATI Industrial Automation, NC, USA) with a precision grip of thumb and index finger, lift it, and hold it as steady as possible in a certain height and position (indexed by a marker) for 20 s. The elbow of the task-performing arm was not allowed to be placed on the table, but had to be held in the air. The non-task-performing hand was to be placed on the thigh. Start and end of a trial were signaled by acoustic cues. Prior to the first trial, the task was demonstrated by the examiner, and the subjects were supposed to familiarize themselves with the haptics and weight of the grip device. The timing of the three subsequent trials was subject-paced, with breaks of usually a few seconds in between trials. Additional details regarding the apparatus can be found in Reilmann et al. (24). Supplementary Figure 1 displays a typical example of a raw signal recorded during a grasping and lifting trial, together with depictions of outcome measures which can be derived from the raw signal.

In case of grasping and lifting, the outcomes “lifting position-index mean (deg/s)” (LFPIMN; the means of the absolute values of the derivatives of the x, y, and z channels were computed during the static holding phase and summed to create the index) and “lifting orientation-index mean (cm/s)” [LFOIMN; the means of the absolute values of the derivatives of the roll, pitch, and yaw channels were summed and defined as the index (24)] were analyzed; both outcomes are measures of involuntary movement.

Regarding Q-Motor speeded finger tapping, the task was to tap as quickly as possible for 10 s on a force transducer (Mini-40, ATI Industrial Automation, NC, USA) with the elongated index finger. The task-performing hand was placed on a hand rest. The non-task-performing hand was to be placed on the thigh. Start and end of a trial were signaled by acoustic cues. Prior to the first trial, the task was demonstrated by the examiner, and the subjects were supposed to shortly try the task to familiarize themselves with the setup. The timing of the three subsequent trials was subject-paced, with breaks of usually a few seconds in between trials. Additional details regarding the apparatus can be found in Bechtel et al. (3). Supplementary Figure 2 displays a typical example of a raw signal excerpt recorded during a speeded finger tapping trial, together with depictions of outcome measures which can be derived from the raw signal.

In case of speeded finger tapping, the outcomes “tapping frequency mean (Hz)” (TSFRMN-a measure of speed), “tap inter-onset interval mean (s)” (TSIOMN-a measure of speed), and “tap inter-onset interval standard deviation (SD) (s)” (TSIOSD-a measure of irregularity) were analyzed.

As an aside, tapping frequency and mean tap inter-onset interval are related variables that both reflect tapping speed, and there may be no unique extra information in tap inter-onset intervals compared to frequencies. However, tap inter-onset intervals are-like tap durations, tap forces, and inter-tap intervals (which were not reported here)-part of a variable family that describes the *gestalt* and characteristics of individual taps, while the tapping frequency provides a much more general measure of a complete trial. Frequencies are more comprehensible, while inter-onset intervals potentially yield more sensitivity and insight in individual tapping properties.

Pseudonymized data were transferred to the Q-Motor team at the George-Huntington-Institute Münster, where the data were processed, quality-controlled, and pre-analyzed by trained personnel in the following steps: (i) rejection of compromised trials; (ii) baseline filtering of trials; (iii) averaging across valid trials within hand; (iv) compression of averaged signals into point measures of central tendency and/or variability. During quality control, trials were excluded for example if the data contained sensor errors/artifacts (e.g., gauge saturation), or if trials were not recorded according to the protocol (e.g., participants had touched sensors too late or too early). In about 85% of cases, three valid trials could be averaged. In about 98% of cases, at least two valid trials could be averaged. Further (i.e., statistical) analyses were conducted at the BiDirect Study site.

The Purdue Pegboard test examines the ability to manipulate small pins (“pegs”) between the distal parts of the fingers, requiring quick, precise, and fine inter-digital movements. The Purdue Pegboard test was also performed with each hand separately; for each hand, one trial was conducted. The task was to place as many pins as possible into the board, one below the other, within 30 s. Prior to each trial, the subjects were supposed to practice with three pins. The Pegboard data were analyzed at the BiDirect Study site.

In case of Purdue Pegboard testing, the number of pins placed within 30 s (PEGB) was analyzed.

Each outcome was usually available for left and right hands. The selection of outcomes to be analyzed was exclusively theory-driven; no variable selection algorithms were applied. An overview regarding the motor outcomes is given in Table 1.

**Table 1:** Overview of motor outcomes.

**Outcome**	**Abbreviation**	**Hand**	**Task**	**Unit**	**Domain**	**Better performance**
Frequency, mean	TSFRMNHL	Left	Speeded tapping	Hz	Speed	Higher values (higher speed)
Frequency, mean	TSFRMNHR	Right	Speeded tapping	Hz	Speed	Higher values (higher speed)
Inter-onset interval, mean	TSIOMNHL	Left	Speeded tapping	s	Speed	Lower values (higher speed)
Inter-onset interval, mean	TSIOMNHR	Right	Speeded tapping	s	Speed	Lower values (higher speed)
Inter-onset interval, SD	TSIOSDHL	Left	Speeded tapping	s	Irregularity	Lower values (less irregularity)
Inter-onset interval, SD	TSIOSDHR	Right	Speeded tapping	s	Irregularity	Lower values (less irregularity)
Position-index, mean	LFPIMNHL	Left	Grasping and lifting	cm/s	Involuntary movement	Lower values (less involuntary movement)
Position-index, mean	LFPIMNHR	Right	Grasping and lifting	cm/s	Involuntary movement	Lower values (less involuntary movement)
Orientation-index, mean	LFOIMNHL	Left	Grasping and lifting	deg/s	Involuntary movement	Lower values (less involuntary movement)
Orientation-index, mean	LFOIMNHR	Right	Grasping and lifting	deg/s	Involuntary movement	Lower values (less involuntary movement)
Number of pins placed	PEGBHL	Left	Pegboard	pins/30 s	Dexterity	Higher values (more pins placed)
Number of pins placed	PEGBHR	Right	Pegboard	pins/30 s	Dexterity	Higher values (more pins placed)

### Assessment of handedness in the BiDirect Study

The BiDirect Study did not employ a handedness inventory. Rather, participants were asked (at the first and second visits) whether they were right-handed, left-handed, or else. Participants who answered consistently were classified as either right-handers or left-handers, respectively. The handedness of the remaining participants was classified as “unclear.” Most of the results presented in the current article are restricted to the right-handed participants, who constituted by far the largest subgroup.

### Selection of explanatory variables for analysis

Explanatory variables of primary interest were age and sex. Moreover, we considered potential mediating effects of educational attainment (number of years in full-time education; continuous), depressive symptoms in the past seven days [Center for Epidemiologic Studies Depression Scale (CES-D) score (51); continuous], alcohol consumption in the past seven days (average consumption in grams/day; continuous), and smoking status (current vs. former vs. never smoker; categorical). Additionally, we investigated possible effects of different examiners.

### Analysis details

The data were analyzed with R (52) using RStudio Desktop (RStudio Inc., Boston, MA, USA) in several subsequent steps.

**Figure 1:**
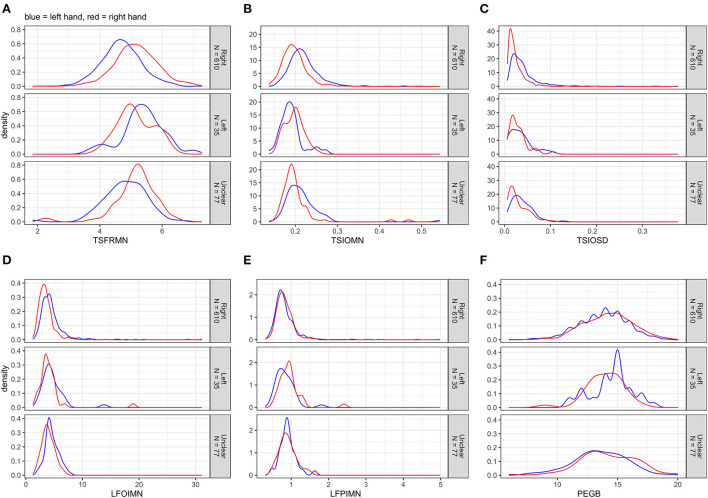
**(A–F)** Distributions of the non-transformed motor outcomes for left and right hands separately, stratified by handedness.

First, we computed univariate multiple linear (Gaussian) regression analyses, modeling the motor outcomes separately. Depending on the distribution of an outcome (Figure 1), we used either non-transformed or log_10_-transformed data [see e.g., (30)]. Model 1 included the explanatory variables of interest, i.e., age and sex, and the adjusted variable examiner; this model estimated total effects of age and sex. Model 2 included all explanatory variables (i.e., additionally education years, CES-D score, alcohol consumption, and smoking status); this model approximated direct effects of age and sex. The presumed causal relationships among the explanatory and motor outcome variables are illustrated in Supplementary Figure 3.

All statistical analyses were computed on the subgroup of right-handed subjects. The significance threshold was set to alpha = 0.05. All analyses were cross-sectional and should be regarded as exploratory.

## Results

Table 2 displays descriptive statistics with respect to explanatory variables used in the present study, stratified by handedness. Supplementary Figure 4 depicts univariate distributions of explanatory variables, stratified by handedness.

**Table 2:** Descriptive statistics for the participants stratified by handedness.

**Explanatory variable**	**Right-handed (*N* = 610)**	**Left-handed (*N* = 35)**	**Handedness unclear (*N* = 77)**
Age [mean (SD) (years)]	56.03 (7.89)	53.60 (8.77)	56.37 (8.80)
Sex [male vs. female; *N* (%)]	286 (46.9%) vs. 324 (53.1%)	23 (65.7%) vs. 12 (34.3%)	41 (53.2%) vs. 36 (46.8%)
Education years [mean (SD)]	14.84 (2.67)	15.54 (2.70)	15.27 (2.82)
Average alcohol consumption in past 7 days [mean (SD) (grams/day)]	14.64 (18.84)	14.20 (13.61)	15.57 (20.76)
Smoking status [Never vs. former vs. current; *N* (%)]	257 (42.2%) vs. 239 (39.2%) vs. 113 (18.6%)	18 (51.4%) vs. 14 (40.0%) vs. 3 (8.6%)	39 (50.6%) vs. 26 (33.8%) vs. 12 (15.6%)
Depressive symptoms [CES-D* score; mean (SD)]	9.17 (7.55)	10.40 (6.33)	8.53 (6.18)

*Center for Epidemiologic Studies Depression Scale.

The participants were mostly middle-aged to elderly, and the sex ratio was well-balanced with only slightly more women than men. Education years were bi-modally distributed (with peaks around 13 and 18 years of education, respectively), most participants had consumed no or little alcohol and had reported rather few depressive symptoms in the past week and were either never or former smokers. The numbers of examinations conducted by the (overall 12) different examiners varied considerably (minimum = 1, maximum = 261). The vast majority of participants (84.4%) was right-handed.

Figure 1 displays the distributions of the non-transformed motor outcomes for left and right hands separately, stratified by handedness. The tapping frequency mean (TSFRMN) and the Pegboard number of pins placed (PEGB) were normally distributed; the remaining outcomes were right-skewed.

**Figure 2:**
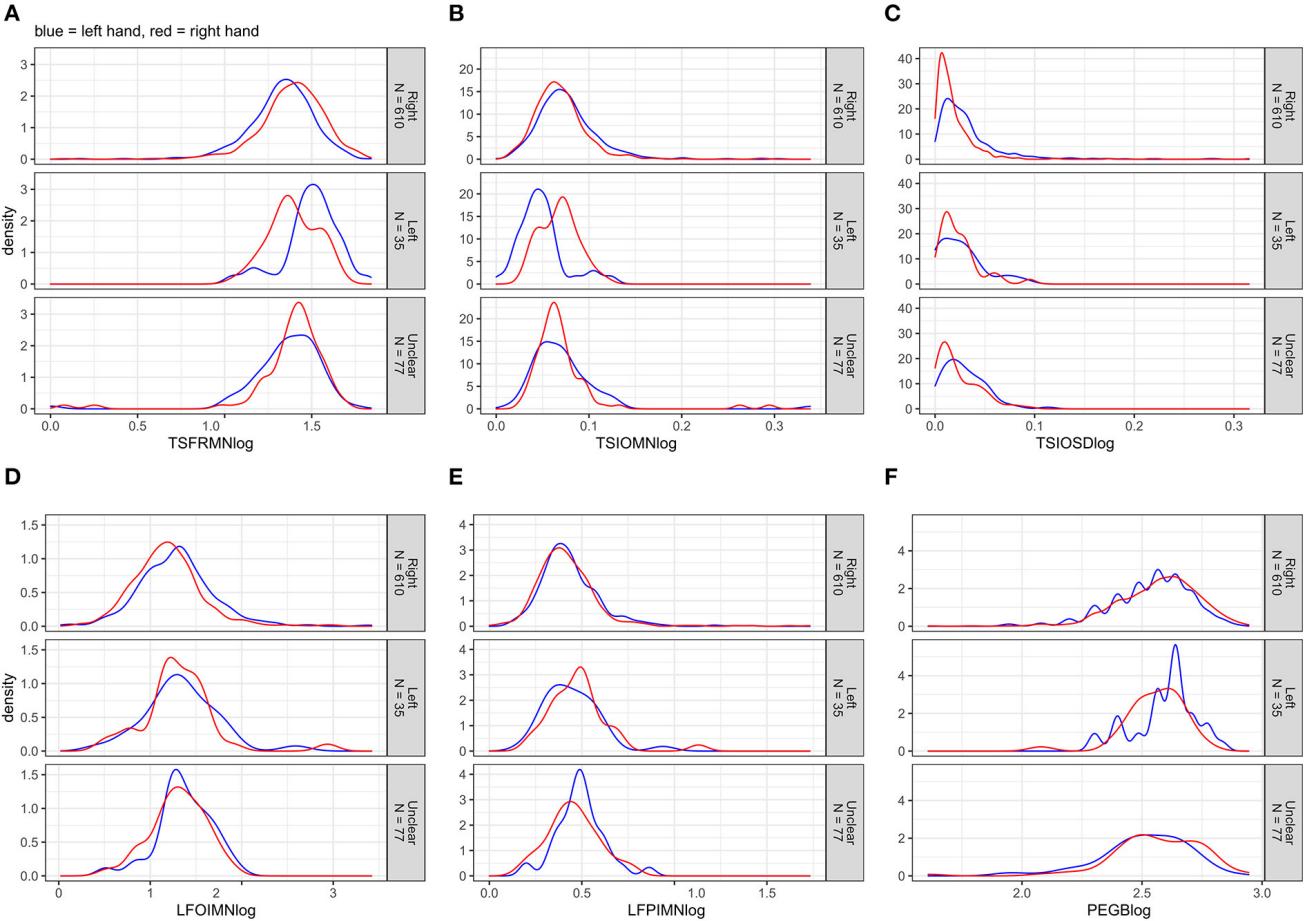
**(A–F)** Distributions of the log_10_-transformed motor outcomes for left and right hands separately, stratified by handedness.

Figure 2 displays the distributions of the outcomes after log_10_-transformation. In cases of log lifting position-index mean (LFPIMNlog) and log lifting orientation-index mean (LFOIMNlog), the distributions changed noticeably toward normality; this was not the case for log tap inter-onset interval mean (TSIOMNlog) and log tap inter-onset interval SD (TSIOSDlog).

Table 3 displays measures of central tendency and variability for the non-transformed outcomes in left and right hands separately, stratified by test, age category, and handedness. Table 4 displays these measures stratified by test, sex, and handedness. In all outcomes and across the age categories and the sexes, right-handers tended to perform better with their right hand, and left-handers tended to perform better with their left hand.

**Table 3:** Measures of central tendency and variability for the (non-transformed) motor outcomes in left and right hands, stratified by test, age category, and handedness.

**Q-Motor speeded finger tapping outcome**	**Frequency, mean, left hand**			**Frequency, mean, right hand**			**Inter-onset interval, mean, left hand**			**Inter-onset interval, mean, right hand**			**Inter-onset interval, sd, left hand**			**Inter-onset interval, sd, right hand**		
Age category (years)	>37 to 50	>50 to 60	>60 to 70	>37 to 50	>50 to 60	>60 to 70	>37 to 50	>50 to 60	>60 to 70	>37 to 50	>50 to 60	>60 to 70	>37 to 50	>50 to 60	>60 to 70	>37 to 50	>50 to 60	>60 to 70
**Right-handed**																		
Mean	4.91	4.77	4.45	5.34	5.24	4.97	0.21	0.21	0.23	0.19	0.19	0.20	0.04	0.04	0.04	0.03	0.03	0.02
SD	0.71	0.61	0.59	0.80	0.66	0.62	0.04	0.03	0.03	0.04	0.03	0.03	0.03	0.03	0.04	0.03	0.03	0.02
Median	4.85	4.77	4.49	5.28	5.25	4.93	0.21	0.21	0.22	0.19	0.19	0.20	0.03	0.03	0.03	0.02	0.02	0.02
MAD	0.59	0.58	0.60	0.65	0.68	0.62	0.03	0.03	0.03	0.02	0.02	0.02	0.02	0.02	0.02	0.01	0.01	0.01
N	157	223	230	157	223	230	157	223	230	157	223	230	157	223	230	157	223	230
**Left-handed**																		
Mean	5.43	5.39	5.25	4.95	5.28	5.18	0.19	0.19	0.19	0.21	0.19	0.19	0.04	0.04	0.03	0.03	0.03	0.03
SD	0.63	0.80	0.56	0.63	0.56	0.48	0.02	0.03	0.02	0.03	0.02	0.02	0.03	0.03	0.02	0.03	0.02	0.02
Median	5.34	5.58	5.22	5.02	5.23	5.03	0.19	0.18	0.19	0.20	0.19	0.20	0.04	0.03	0.04	0.02	0.02	0.02
MAD	0.38	0.47	0.34	0.84	0.63	0.21	0.01	0.01	0.01	0.04	0.02	0.01	0.02	0.01	0.03	0.01	0.01	0.01
N	11	15	9	11	15	9	11	15	9	11	15	9	11	15	9	11	15	9
**Handedness unclear**																		
Mean	4.66	4.99	4.78	5.13	5.32	5.01	0.23	0.20	0.22	0.20	0.19	0.21	0.04	0.04	0.04	0.03	0.03	0.03
SD	0.78	0.53	0.74	0.80	0.53	0.74	0.08	0.02	0.03	0.06	0.02	0.05	0.02	0.02	0.02	0.03	0.02	0.02
Median	4.76	5.25	4.84	5.23	5.33	5.10	0.21	0.19	0.21	0.19	0.19	0.20	0.03	0.03	0.03	0.02	0.02	0.02
MAD	0.50	0.67	0.92	0.53	0.56	0.39	0.02	0.03	0.03	0.02	0.02	0.01	0.02	0.03	0.02	0.01	0.02	0.02
N	20	25	32	20	25	32	20	25	32	20	25	32	20	25	32	20	25	32
**Q-Motor grasping and lifting outcome**	**Position index, mean, left hand**			**Position index, mean, right hand**			**Orientation index, mean, left hand**			**Orientation index, mean, right hand**								
Age category (years)	>37 to 50	>50 to 60	>60 to 70	>37 to 50	>50 to 60	>60 to 70	>37 to 50	>50 to 60	>60 to 70	>37 to 50	>50 to 60	>60 to 70						
**Right-handed**
Mean	0.83	0.81	0.84	0.83	0.83	0.89	4.40	4.47	4.60	3.54	3.52	3.84						
**Purdue Pegboard outcome**	**Number of pins placed, left hand**			**Number of pins placed, right hand**														
Age category (years)	>37 to 50	>50 to 60	>60 to 70	>37 to 50	>50 to 60	>60 to 70												
**Right-handed**																		
Mean	14.76	13.99	13.04	15.19	14.31	13.46												
SD	1.84	1.90	1.88	1.89	1.96	1.95												
Median	15.00	14.00	13.00	15.00	14.00	14.00												
MAD	1.48	1.48	1.48	1.48	1.48	1.48												
N	157	223	230	157	223	230												
**Left-handed**																		
Mean	14.82	15.13	13.00	13.73	14.07	14.11												
SD	1.54	1.41	1.58	1.95	1.10	1.62												
Median	15.00	15.00	13.00	14.00	14.00	14.00												
MAD	1.48	1.48	1.48	1.48	1.48	1.48												
N	11	15	9	11	15	9												
**Handedness unclear**																		
Mean	14.00	13.80	12.97	15.10	14.36	13.44												
SD	1.97	2.12	2.21	2.27	2.10	2.41												
Median	14.50	14.00	13.00	15.00	14.00	13.00												
MAD	2.22	1.48	1.48	2.97	2.97	1.48												
N	20	25	32	20	25	32												

**Table 4:** Measures of central tendency and variability for the (non-transformed) motor outcomes in left and right hands, stratified by test, sex, and handedness.

**Q-Motor speeded finger tapping outcome**	**Frequency, mean, left hand**		**Frequency, mean, right hand**		**Inter-onset interval, mean, left hand**		**Inter-onset interval, mean, right hand**		**Inter-onset interval, sd, left hand**		**Inter-onset interval, sd, right hand**	
Sex	Male	Female	Male	Female	Male	Female	Male	Female	Male	Female	Male	Female
**Right-handed**											
Mean	4.89	4.50	5.40	4.95	0.21	0.23	0.19	0.21	0.04	0.04	0.02	0.03
SD	0.69	0.57	0.71	0.62	0.04	0.03	0.03	0.03	0.04	0.03	0.01	0.03
Median	4.89	4.52	5.38	4.97	0.21	0.22	0.19	0.20	0.03	0.03	0.02	0.02
MAD	0.61	0.52	0.72	0.54	0.03	0.02	0.03	0.02	0.02	0.02	0.01	0.01
N	286	324	286	324	286	324	286	324	286	324	286	324
**Left-handed**											
Mean	5.57	4.97	5.27	4.91	0.18	0.21	0.19	0.21	0.03	0.04	0.03	0.03
SD	0.58	0.71	0.57	0.48	0.02	0.03	0.02	0.02	0.02	0.03	0.02	0.02
Median	5.56	5.09	5.13	4.88	0.18	0.20	0.20	0.21	0.03	0.04	0.02	0.03
MAD	0.50	0.67	0.65	0.42	0.02	0.02	0.02	0.02	0.02	0.03	0.01	0.02
N	23	12	23	12	23	12	23	12	23	12	23	12
**Handedness unclear**											
Mean	5.09	4.50	5.29	4.97	0.20	0.23	0.19	0.21	0.03	0.04	0.02	0.04
SD	0.58	0.69	0.66	0.72	0.02	0.06	0.05	0.04	0.02	0.03	0.01	0.03
Median	5.17	4.52	5.30	5.07	0.20	0.22	0.19	0.20	0.03	0.04	0.02	0.03
MAD	0.54	0.59	0.52	0.52	0.02	0.03	0.02	0.02	0.02	0.02	0.01	0.03
N	41	36	41	36	41	36	41	36	41	36	41	36
**Q-Motor grasping and lifting outcome**	**Position index, mean, left hand**		**Position index, mean, right hand**		**Orientation index, mean, left hand**		**Orientation index, mean, right hand**					
Sex	Male	Female	Male	Female	Male	Female	Male	Female				
**Right-handed**											
Mean	0.86	0.79	0.91	0.81	4.85	4.19	3.89	3.43				
SD	0.35	0.24	0.44	0.26	2.95	1.57	2.08	1.52				
Median	0.80	0.75	0.85	0.76	4.30	3.95	3.53	3.19				
MAD	0.22	0.15	0.18	0.16	1.46	1.04	1.11	0.85				
N	286	324	286	324	286	324	286	324				
**Left-handed**											
Mean	0.89	0.71	1.01	0.84	5.01	3.88	4.64	3.43				
SD	0.27	0.15	0.37	0.16	2.25	1.28	3.30	0.97				
Median	0.85	0.65	0.95	0.80	4.37	3.93	4.16	3.33				
MAD	0.23	0.11	0.19	0.19	1.15	0.85	0.95	0.39				
N	23	12	23	12	23	12	23	12				
**Handedness unclear**											
Mean	0.92	0.90	0.92	0.89	4.62	4.61	4.04	3.90				
SD	0.23	0.21	0.24	0.22	1.26	1.07	1.26	0.91				
Median	0.89	0.88	0.92	0.84	4.41	4.27	3.91	3.79				
MAD	0.23	0.12	0.23	0.17	0.98	0.83	1.00	0.96				
N	41	36	41	36	41	36	41	36				
**Purdue Pegboard outcome**	**Number of pins placed, left hand**		**Number of pins placed, right hand**									
Sex	Male	Female	Male	Female								
**Right-handed**											
Mean	13.28	14.32	13.67	14.70								
SD	2.04	1.81	2.06	1.93								
Median	13.00	14.00	14.00	15.00								
MAD	1.48	1.48	1.48	1.48								
N	286	324	286	324								
**Left-handed**											
Mean	14.83	13.83	14.04	13.83								
SD	1.59	1.80	1.33	1.85								
Median	15.00	14.00	14.00	14.00								
MAD	1.48	2.22	1.48	1.48								
N	23	12	23	12								
**Handedness unclear**											
Mean	13.22	13.83	13.27	15.19								
SD	2.15	2.12	2.24	2.05								
Median	13.00	14.00	13.00	15.50								
MAD	1.48	1.48	1.48	2.22								
N	41	36	41	36								

**Figure 3:**
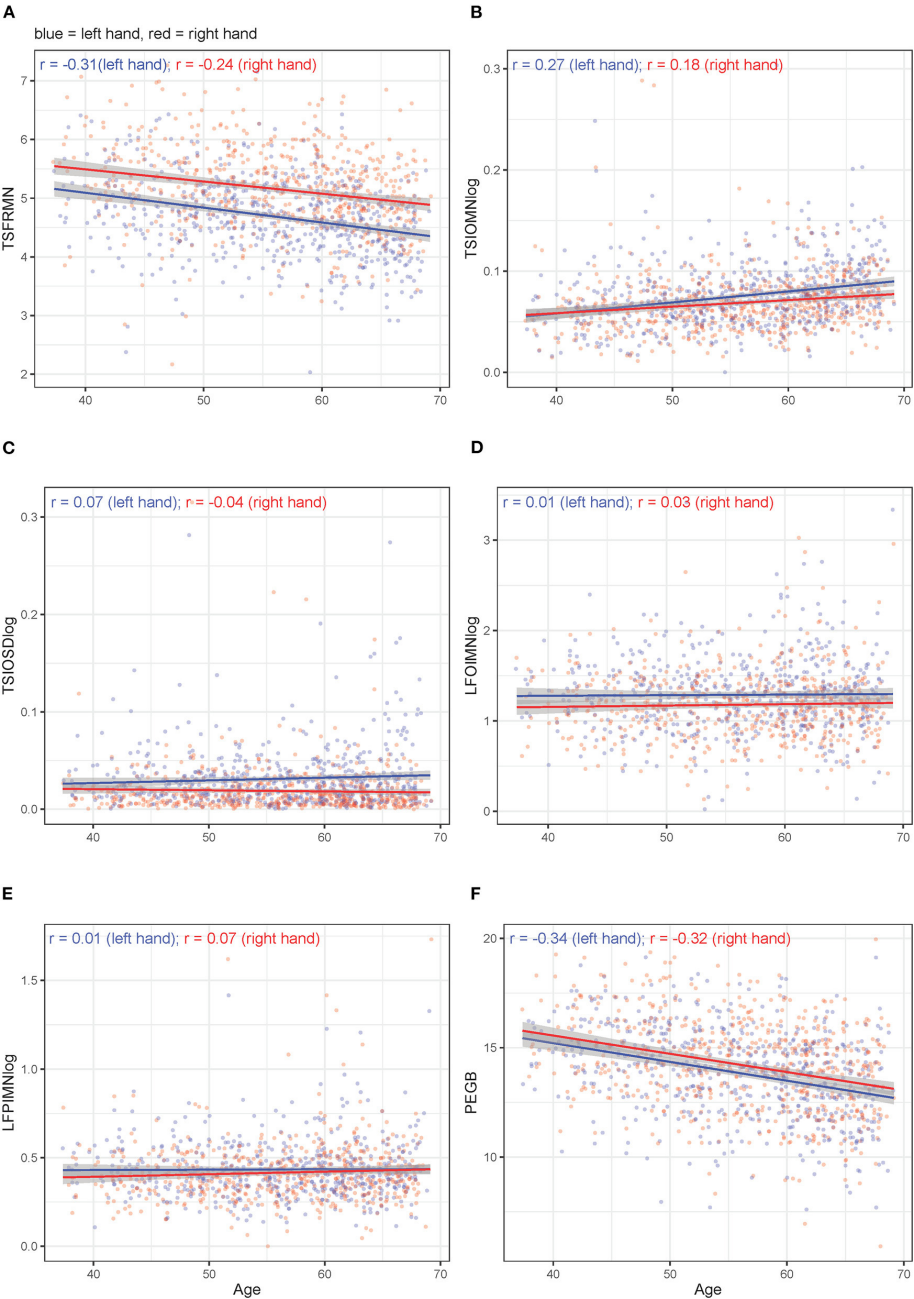
**(A–F)** Unadjusted, bivariate associations between age and the motor outcomes in right-handed participants for left and right hands separately.

**Figure 4:**
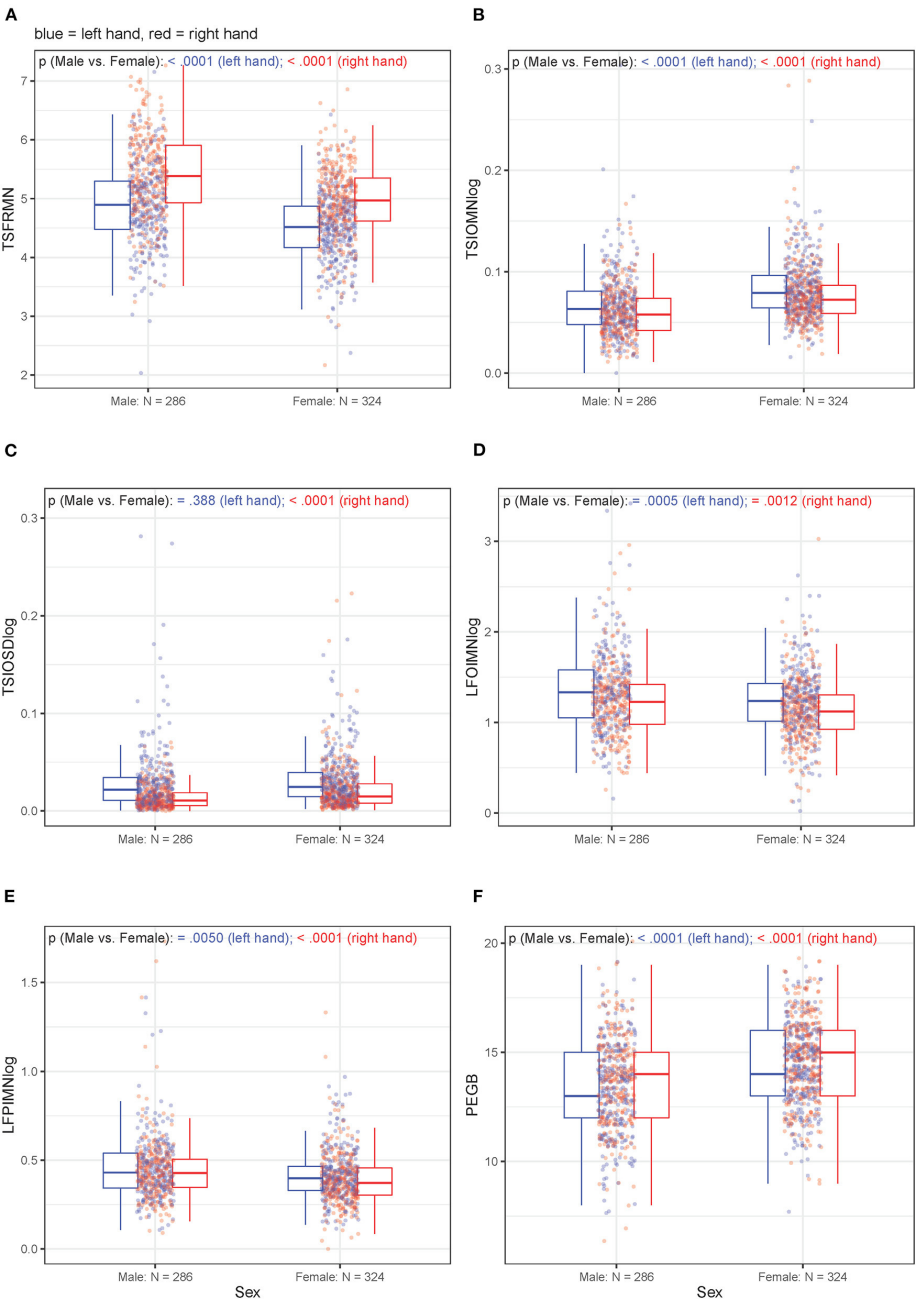
**(A–F)** Unadjusted, bivariate associations between sex and the motor outcomes in right-handed participants for left and right hands separately.

Unadjusted, bivariate associations between age or sex and motor outcome variables (either non-transformed or log_10_-transformed, depending on the original distribution) for the right-handed participants are displayed in Figures 3, 4, respectively. Supplementary Figure 5 through 9 hold analogous displays for the remaining explanatory variables. With regard to age, the visually most noticeable associations were with tapping frequency mean (TSFRMN), log tap inter-onset interval mean (TSIOMNlog), and Pegboard number of pins placed (PEGB). With regard to sex, the visually most noticeable associations were with tapping frequency mean (TSFRMN), log tap inter-onset interval mean (TSIOMNlog), log lifting orientation-index mean (LFOIMNlog), and Pegboard number of pins placed (PEGB).

Table 5 summarizes (Pearson product-moment) correlation coefficients between all motor outcomes (either non-transformed or log_10_-transformed, depending on the original distribution) for the right-handed subjects, stratified by task-performing hand.

**Table 5:** Correlations between Q-Motor and Pegboard outcomes for the right-handed participants, stratified by task-performing hand.

	**Tapping frequency, mean**	**Log tap inter- onset interval, mean**	**Log tap inter- onset interval, SD**	**Log lifting orientation- index, mean**	**Log lifting position- index mean**
**Left-hand data**					
Log tap inter-onset interval, mean	−0.97*	—	—	—	—
Log tap inter-onset interval, SD	−0.38*	0.44*	—	—	—
Log lifting orientation- index, mean	0.04	−0.01	0.01	—	—
Log lifting position- index, mean	−0.07	0.09*	0.03	0.82*	—
Pegboard number of pins placed	0.22*	−0.21*	−0.02	−0.18*	−0.25*
**Right-hand data**					
Log tap inter-onset interval, mean	−0.95*	—	—	—	—
Log tap inter-onset interval, SD	−0.26*	0.40*	—	—	—
Log lifting orientation- index, mean	0.07	−0.05	−0.01	—	—
Log lifting position- index, mean	−0.02	0.03	−0.02	0.81*	—
Pegboard number of pins placed	0.18*	−0.14*	0.05	−0.20*	−0.24*

*Significant at p ≤ 0.05.

Supplementary Figure 10 displays associations between Purdue Pegboard and Q-Motor outcomes (either non-transformed or log_10_-transformed). All such associations were positive, i.e., better performance in Pegboard was generally associated with better performance in Q-Motor. There was no correlation between Pegboard number of pins placed (PEGB) and tap inter-onset interval SD (TSIOSD). The remaining Pegboard - Q-Motor correlations were only weak.

Except for tap inter-onset SD-left hand and log tap inter-onset SD-left hand (in both models 1 and 2), all multiple linear regression analyses were significant. Adjusted *R*^2^ values of significant models were in the range between 3.69% (lifting position-index mean-right hand) and 18.54% (Pegboard number of pins placed-left hand) for model 1 (Table 6); for model 2, the range was from 3.91% (tap inter-onset SD-right hand) to 21.09% (Pegboard number of pins placed-left hand; Table 7).

**Table 6:** Model 1 quality metrics.

	**Scale**	**Adjusted *R^2^***	***P*-value**	**Df**	***N***
**Speeded finger tapping**					
Frequency, mean, left hand	Non-transformed	0.178	0.00000	12	563
Frequency, mean, right hand	Non-transformed	0.158	0.00000	12	559
Log inter-onset interval, mean, left hand	Log_10_	0.123	0.00000	12	563
Log inter-onset interval, mean, right had	Log_10_	0.114	0.00000	12	559
Log inter-onset interval, SD, left hand	Log_10_	0.010	0.12264	12	563
Log inter-onset interval, SD, right hand	Log_10_	0.043	0.00027	12	559
**Grasping and lifting**					
Log position-index, mean, left hand	Log_10_	0.060	0.00002	12	513
Log position-index, mean, right hand	Log_10_	0.047	0.00022	12	525
Log orientation-index, mean, left hand	Log_10_	0.050	0.00015	12	514
Log orientation-index, mean, right hand	Log_10_	0.067	0.00000	12	525
**Purdue Pegboard**					
Number of pins placed, left hand	Non-transformed	0.185	0.00000	12	569
Number of pins placed, right hand	Non-transformed	0.150	0.00000	12	569

**Table 7:** Model 2 quality metrics.

	**Scale**	**Adjusted *R^2^***	***P*-value**	**Df**	***N***
**Speeded finger tapping**					
Frequency, mean, left hand	Non-transformed	0.199	0.00000	17	559
Frequency, mean, right hand	Non-transformed	0.173	0.00000	17	556
Log inter-onset interval, mean, left hand	Log_10_	0.156	0.00000	17	559
Log inter-onset interval, mean, right hand	Log_10_	0.125	0.00000	17	556
Log inter-onset interval, SD, left hand	Log_10_	0.007	0.21287	17	559
Log inter-onset interval, SD, right hand	Log_10_	0.040	0.00157	17	556
**Grasping and lifting**					
Log position-index, mean, left hand	Log_10_	0.111	0.00000	17	509
Log position-index, mean, right hand	Log_10_	0.084	0.00000	17	521
Log orientation-index, mean, left hand	Log_10_	0.112	0.00000	17	510
Log orientation-index, mean, right hand	Log_10_	0.130	0.00000	17	521
**Purdue Pegboard**					
Number of pins placed, left hand	Non-transformed	0.210	0.00000	17	565
Number of pins placed, right hand	Non-transformed	0.183	0.00000	17	565

Moreover, adjusted *R*^2^ values (which take the number of terms in the model into account) were usually higher in model 2 compared to model 1, indicating that model 2 tended to be a better fit to the data (Tables 6, 7).

With regard to individual explanatory variables from model 1 (including age, sex, and examiner), the results demonstrated significant effects of age for tapping frequency mean (left + right hands), log tap inter-onset interval mean (left + right hands), and Pegboard number of pins placed (left + right hands). In each case, more advanced age was associated with decreased performance. Significant effects of sex were found for tapping frequency mean (left + right hands; female sex was associated with decreased performance), log tap inter-onset interval mean (left + right hands; decreased performance in females), log tap inter-onset interval SD (right hand; decreased performance in females), log lifting position-index mean (left + right hands; increased performance in females), log lifting orientation-index mean (left + right hands; increased performance in females), and Pegboard number of pins placed (left + right hands; increased performance in females). Significant effects of examiner were found for log lifting position-index mean (left + right hands) and log lifting orientation-index mean (left + right hands; Table 8).

**Table 8:** Effects of individual explanatory variables, model 1.

	**Explanatory variable**	**Df**	***F*-value**	***P*-value**
**Outcome (speeded finger tapping)**				
Frequency, mean,	Age	1	46.78845	0.00000
left hand	Sex	1	58.7728	0.00000
	Examiner	10	1.60046	0.10281
Frequency, mean,	Age	1	28.23143	0.00000
right hand	Sex	1	70.89185	0.00000
	Examiner	10	1.11749	0.3465
Log inter-onset interval,	Age	1	35.37066	0.00000
mean, left hand	Sex	1	37.53526	0.00000
	Examiner	10	0.96312	0.4747
Log inter-onset interval,	Age	1	13.91822	0.00021
mean, right hand	Sex	1	52.80957	0.00000
	Examiner	10	1.11183	0.35078
Log inter-onset interval,	Age	1	1.36885	0.24252
SD, left hand	Sex	1	0.77085	0.38034
	Examiner	10	1.53217	0.12419
Log inter-onset interval,	Age	1	1.79482	0.1809
SD, right hand	Sex	1	16.78345	0.00005
	Examiner	10	1.59312	0.10497
**Outcome (grasping and lifting)**				
Log position-index,	Age	1	0.18096	0.67073
mean, left hand	Sex	1	6.41986	0.01159
	Examiner	10	3.72484	0.00008
Log position-index,	Age	1	3.27031	0.07113
mean, right hand	Sex	1	14.05219	0.0002
	Examiner	10	2.0274	0.0289
Log orientation-index,	Age	1	0.0881	0.76673
mean, left hand	Sex	1	10.57954	0.00122
	Examiner	10	2.63605	0.00389
Log orientation-index,	Age	1	0.76455	0.38232
mean, right hand	Sex	1	8.5653	0.00358
	Examiner	10	3.85282	0.00005
**Outcome (Purdue Pegboard)**				
Number of pins	Age	1	71.98546	0.00000
placed, left hand	Sex	1	44.17509	0.00000
	Examiner	10	1.84567	0.05031
Number of pins	Age	1	54.97657	0.00000
placed, right hand	Sex	1	37.96445	0.00000
	Examiner	10	1.40193	0.17553

Regarding effects of individual explanatory variables from model 2 (additionally including education, alcohol consumption, smoking status, and depressive symptoms), the results demonstrated significant effects of age for tapping frequency mean (left + right hands), log tap inter-onset interval mean (left + right hands), and Pegboard number of pins placed (left + right hands). In each case, advanced age was associated with decreased performance. Significant effects of sex were found for tapping frequency mean (left + right hands; decreased performance in females), log tap inter-onset interval mean (left + right hands; decreased performance in females), log tap inter-onset interval SD (right hand; decreased performance in females), log lifting position-index mean (left + right hands; increased performance in females), log lifting orientation-index mean (left + right hands; increased performance in females), and Pegboard number of pins placed (left + right hands; increased performance in females). Significant effects of education were found for tapping frequency mean (left + right hands), log tap inter-onset interval mean (left + right hands), log lifting position-index mean (left hand), and Pegboard number of pins placed (left + right hands). In each case, more education years were associated with increased performance. A significant effect for alcohol consumption was found for log lifting orientation-index mean (left hand); more alcohol consumed was associated with decreased performance. Significant effects for smoking status were found for log position-index mean (left + right hands; current smoking was associated with decreased performance compared to never smoking), log lifting orientation index mean (left + right hands; current smoking was associated with decreased performance compared to never smoking), and Pegboard number of pins placed (left + right hands; both current and former smoking were associated with decreased performance compared to never smoking). A significant effect of depressive symptoms was found for Pegboard number of pins placed (left hand; more depressive symptoms were associated with decreased performance). Significant effects of examiner were found for tapping frequency mean (left hand), log lifting position-index mean (left + right hands), and log lifting orientation-index mean (left + right hands; Table 9).

**Table 9:** Effects of individual explanatory variables, model 2.

	**Explanatory variable**	**Df**	***F*-value**	***P*-value**
**Outcome (speeded finger tapping)**				
Frequency, mean,	Age	1	41.21468	0.00000
left hand	Sex	1	52.79897	0.00000
	Education years	1	6.58897	0.01053
	CES-D score	1	1.8691	0.17215
	Alcohol consumption	1	2.11959	0.14601
	Smoking status	2	0.20102	0.81795
	Examiner	10	1.90666	0.04186
Frequency, mean,	Age	1	24.23902	0.00000
right hand	Sex	1	60.14618	0.00000
	Education years	1	8.58415	0.00353
	CES-D score	1	2.57215	0.10935
	Alcohol consumption	1	1.23594	0.26675
	Smoking status	2	0.57343	0.56393
	Examiner	10	1.09221	0.36593
Log inter-onset interval,	Age	1	33.49364	0.00000
mean, left hand	Sex	1	38.13605	0.00000
	Education years	1	6.09867	0.01384
	CES-D score	1	1.27099	0.26008
	Alcohol consumption	1	2.55477	0.11055
	Smoking status	2	0.38423	0.68116
	Examiner	10	1.34601	0.2025
Log inter-onset interval,	Age	1	11.84349	0.00062
mean, right hand	Sex	1	44.3634	0.00000
	Education years	1	5.25529	0.02227
	CES-D score	1	2.65739	0.10366
	Alcohol consumption	1	1.1149	0.29149
	Smoking status	2	0.41981	0.65739
	Examiner	10	1.10338	0.35728
Log inter-onset interval,	Age	1	0.48583	0.48609
SD, left hand	Sex	1	0.7968	0.37245
	Education years	1	0.44603	0.50451
	CES-D score	1	0.07131	0.78954
	Alcohol	1	0.0009	0.97608
	consumption			
	Smoking status	2	1.39076	0.24977
	Examiner	10	1.50386	0.13417
Log inter-onset interval,	Age	1	1.79017	0.18147
SD, right hand	Sex	1	16.37036	0.00006
	Education years	1	0.35323	0.55254
	CES-D score	1	1.35245	0.24537
	Alcohol consumption	1	0.00023	0.98782
	Smoking status	2	0.79747	0.451
	Examiner	10	1.60555	0.10143
**Outcome (grasping and lifting)**				
Log position-index,	Age	1	0.31938	0.57224
mean, left hand	Sex	1	7.36862	0.00687
	Education years	1	4.66636	0.03124
	CES-D score	1	0.07012	0.79128
	Alcohol consumption	1	1.07952	0.29932
	Smoking status	2	10.84659	0.00002
	Examiner	10	3.90602	0.00004
Log position-index,	Age	1	3.58816	0.05877
mean, right hand	Sex	1	13.45946	0.00027
	Education years	1	1.99947	0.15797
	CES-D score	1	0.00144	0.9697
	Alcohol consumption	1	1.17669	0.27855
	Smoking status	2	9.2715	0.00011
	Examiner	10	2.19473	0.01698
Log orientation-index,	Age	1	0.27961	0.59719
mean, left hand	Sex	1	9.57638	0.00208
	Education years	1	2.20638	0.13808
	CES-D score	1	0.08595	0.76952
	Alcohol consumption	1	5.36385	0.02097
	Smoking	2	13.92164	0.00000
	status			
	Examiner	10	2.92959	0.0014
Log orientation-index,	Age	1	1.44047	0.23063
mean, right hand	Sex	1	7.25292	0.00731
	Education years	1	0.32646	0.56801
	CES-D score	1	0.07944	0.77818
	Alcohol consumption	1	3.74149	0.05364
	Smoking status	2	16.94653	0.00000
	Examiner	10	4.30731	0.00001
**Outcome (Purdue Pegboard)**				
Number of pins placed, left hand	Age	1	58.47763	0.00000
	Sex	1	47.76105	0.00000
	Education years	1	4.58328	0.03273
	CES-D score	1	5.90957	0.01538
	Alcohol consumption	1	0.2463	0.61989
	Smoking status	2	3.56169	0.02905
	Examiner	10	1.87621	0.04591
Number of pins placed, right hand	Age	1	42.86278	0.00000
	Sex	1	44.02149	0.00000
	Education years	1	7.33939	0.00696
	CES-D score	1	1.33375	0.24864
	Alcohol consumption	1	0.66693	0.41448
	Smoking status	2	6.60936	0.00146
	Examiner	10	1.33787	0.20664

In the right-handed subjects who were analyzed here, as a general pattern, the models tended to perform better in case of left-hand as compared to right-hand data for tapping frequency mean, tap inter-onset interval mean, lifting position-index mean, and Pegboard number of pins placed. For lifting orientation-index mean, the models tended to perform better in case of right-hand data (Tables 6, 7).

## Discussion

Based on observational, cross-sectional data of middle-aged to elderly, community-dwelling adults from the BiDirect Study (49, 50), the aims of the present analyses were to (i) investigate associations of age and sex with five selected outcomes representing the Q-Motor core tests speeded finger tapping (speed, irregularity) and grasping and lifting (involuntary movements). Furthermore, we (ii) explored effects of the (potentially mediating) variables education, alcohol consumption, smoking status, and depressive symptoms, (iii) explored inter-examiner variability, and (iv) compared the findings regarding the Q-Motor outcomes to findings for the Purdue Pegboard test. Although descriptive findings are presented stratified by handedness, the statistical analyses were restricted to right-handed participants, which constituted the by far largest subgroup.

### Effects of age and sex on Q-Motor speeded finger tapping performance

In the present study, significant effects of age were found for finger tapping speed (tapping frequency mean, left and right hands; log tap inter-onset interval mean, left and right hands), but not with finger tapping irregularity. More advanced age was associated with reduced speed.

Significant effects of sex were found for finger tapping speed (tapping frequency mean, left and right hands; log tap inter-onset interval mean, left and right hands) and finger tapping irregularity (log tap inter-onset interval SD, right hand). Male sex was associated with increased speed and less irregularity.

The age and sex effects on finger tapping speed and irregularity observed here with the specific Q-Motor finger tapping apparatus (electric force-transducer, customized software tool to record the signals) and mode of test administration and data analysis (averaging across three consecutive trials of in each case 10 s duration) basically replicated the findings of numerous previous studies employing a number of different devices, modes of test administration, and sample age and sex distributions [see e.g., (35) for an overview]. Thus, the present findings basically argue in favor of the validity of the Q-Motor speeded finger tapping test. This is further supported by the observation that the overall finger tapping rates (Tables 3, 4) were well in the range of the 4.8 to 5.7 taps per second that was usually reported in the literature. Also, tapping rates were about 10% faster in the preferred compared to the non-preferred hand (cf. Tables 3, 4, right-handers), a finding which has been often reported before [see e.g., (35)]. Moreover, the present findings suggest the feasibility of the Q-Motor speeded finger tapping test for the setting of a large observational study; this is not self-evident, as such studies are typically characterized by less controlled conditions, including e.g., less-specifically trained and more frequently alternating personnel, or limited time to complete many different assessments (53).

Notably, sensitivity of Q-Motor finger tapping speed for the age of subjects was also found in a previous experimental study (*N* = 29) with children and adolescents (30), where younger children tapped slower than older children. However, this study did also find age effects on tapping irregularity (which were not found here in adults), with younger children showing more irregularity than older children. However, the sensitivity of Q-Motor finger tapping speed and irregularity for the sex of subjects found in the present study had *not* been observed by van der Plas et al. in their study with children. Although it appears generally difficult to compare motor performance in children vs. adults, it is interesting to note such similarities and disparities, albeit we acknowledge that the small sample size of their study may also account for the differences.

### Effects of age and sex on Q-Motor grasping and lifting performance

In the present study, there were no significant effects of age on involuntary movement.

Significant effects of sex on involuntary movement were found for both position (log lifting position-index mean, left and right hands) and orientation (log lifting orientation-index mean, left and right hands) indexes. Female sex was associated with less involuntary movement.

Age and sex effects on Q-Motor grasping and lifting outcomes had not yet been systematically investigated in community-dwelling adults. Thus, the present results constitute an important dataset for the design of future studies. Notably, the lack of sensitivity of Q-Motor involuntary movement measures for the age of subjects observed here is in contrast to the results of van der Plas et al. (30) in children and adolescents, where younger children were found to show more involuntary movement with regard to both position as well as orientation compared to older children. This may be explained by maturation of motor coordination in children. Interestingly, this study is the first to detect sensitivity of Q-Motor involuntary movement measures for the sex of subjects (not found by van der Plas et al. in children).

Notably, the mean position and orientation indexes observed here (Tables 3, 4) for the right-handers were well in the range of those reported by Reilmann et al. (24) for their sample of healthy, right-handed control subjects (*N* = 19; 12 females; age range 20 to 63 years).

### Effects of potential mediators on Q-Motor speeded finger tapping performance

In the present study, there were significant effects of education on finger tapping speed (tapping frequency mean, left and right hands; log tap inter-onset interval mean, left and right hands), but not on finger tapping irregularity. More years of education were associated with increased speed.

Previous findings regarding effects of IQ and education on finger tapping performance were somewhat mixed, but there are studies which found that tapping speed was higher with increasing IQ [e.g., (36)] and more education years [e.g., (9, 54)], though effects were comparably smaller than age and sex effects. The education effects on finger tapping speed observed here with the Q-Motor apparatus and mode of test administration replicated the findings of several previous studies using different devices or modes of test administration [e.g., (9)] and hence argue in favor of the validity of Q-Motor speeded finger tapping, as well as its feasibility for large observational studies.

Although typically observed (and while it appears important to register them), it remains unresolved why education effects on finger tapping performance arise in the first place [e.g., (9)]. However, it is reasonable to assume that education is a proxy for a number of underlying person characteristics. For instance, one may speculate that such effects would represent e.g., more motivation or effort, better ability to focus, better task comprehension, better performance strategies, possibly more access to motor or fine motor challenges and exercises, or better health of those comparably higher educated.

There were no significant effects of alcohol consumption, smoking status, or depressive symptoms on finger tapping performance.

### Effects of potential mediators on Q-Motor grasping and lifting performance

In the present study, significant effects of education on involuntary movement were found for position (log lifting position-index mean, left hand): more years of education were associated with less involuntary movement. Given that there are no previous studies, this result is a novel contribution. As discussed above, potential explanations for such education effects currently remain speculative.

Significant effects of smoking status were found for position (log lifting position-index mean, left and right hands) and orientation (log lifting orientation-index mean, left and right hands) indexes: never smoking was associated with less involuntary movement compared to current smoking. These effects seem rather robust, as they are detected in both the position and orientation indexes bilaterally. Effects of smoking status on involuntary movement could theoretically materialize in different ways, among them direct effects of nicotine (or nicotine withdrawal) on the central nervous system, or indirect, possibly accumulating effects of smoking duration and/ or intensity on vascular or nervous system health. Noteworthy, previous studies have found acute effects of nicotine and smoking on *finger tapping* performance [e.g., (44–47)]. A meta-analysis (55) concluded that those studies possibly reflected true nicotine-related performance enhancements in the form of tapping speed increments. Unfortunately, the BiDirect Study did neither record when a currently smoking participant had smoked his last cigarette, nor were there any rules regarding smoking during the waiting periods, so that short-term effects of nicotine on performance could not be quantified. However, given that current smokers tended to perform worse compared to never smokers, while there was no trend for a performance difference between never and former smokers, one may speculate that acute nicotine deprivation may have had an influence. If true, it may be worthwhile to consider or even regulate nicotine consumption/ smoking behavior in the time window preceding a grasping and lifting session. In conclusion, the Q-Motor effects observed seem plausible and may offer an approach to objectively monitoring impact of smoking on motor coordination in population based studies in a sensitive way.

A significant effect of alcohol consumption was found for the orientation index (log lifting orientation-index mean, left hand): more alcohol consumed was associated with more involuntary movement. Similar to effects of smoking status, effects of alcohol consumption on involuntary movement could theoretically materialize in different ways. It appears most likely that the performance disadvantage seen in those who had drunk comparably more alcohol during the past seven days would reflect either cumulative negative effects of regular or extensive drinking on e.g., vascular or nervous system health, or negative effects of acute alcohol deprivation. However, notably these effects were less robust across measures than the effects of nicotine consumption.

There were no significant effects of depressive symptoms on involuntary movement.

### Inter-examiner variability and Q-Motor performance

In the present study, significant effects of examiner were found for finger tapping speed (tapping frequency mean, left hand) and involuntary movement with regard to position (log lifting position-index mean, left and right hands) as well as orientation (log lifting orientation-index mean, left and right hands).

From our point of view, the results regarding effects of examiner are generally difficult to assess. As a matter of fact, the numbers of examinations carried out by different persons varied considerably (from as few as one to as many as 261). Moreover, the distributions of motor outcomes differed noticeably between different examiners (Supplementary Figure 9), even in cases where persons had conducted comparable numbers of examinations. Furthermore, whether or not a categorical explanatory variable becomes significant in a statistical model depends, amongst others, on the choice of reference category; this choice is somewhat arbitrary and can be data-driven or theoretically motivated. For example, while it appears tempting to use the examiner who had conducted by far the most examinations as the reference, with hindsight there is no way of knowing whether or not this specific examiner administered the tests as intended. In the present study, we decided to use an examiner as the reference who had conducted a “typical” number (i.e., 45) of examinations.

Taken together, the results indicate that, if any, effects of examiner would have affected rather involuntary movement, and not so much tapping speed or irregularity. As a consequence, it may be worthwhile to review and possibly even better standardize Q-Motor grasping and lifting instructions, and to sensitize future examiners for certain critical task specifics prone to insufficiently precise participant briefing.

### Relationships between Q-Motor and Purdue Pegboard outcomes

In the present study, with the exception of log tap inter-onset interval SD, there were positive associations of the Q-Motor outcomes with the Purdue Pegboard outcomes for both hands: better performance in Q-Motor was strictly related with better performance in the Purdue Pegboard (Supplementary Figure 10). The correlation coefficients between Q-Motor and Pegboard were of very similar, but only modest magnitude (Table 5), confirming that these tests partly overlap, but also capture rather different aspects of motor capability. The fact that there was no correlation with speeded finger tapping irregularity is not surprising, as this was the only “variability” measure.

The Purdue Pegboard test is a “classical” neuropsychological test that is characterized by e.g., inexpensiveness, relative simplicity of administration, and simplicity of performance evaluation. In the present study, we found significant and plausible effects of age (reduced performance with advancing age), sex (better performance in females), education (better performance with more years of education), smoking status (better performance in never compared to current as well as former smokers), and examiner with Pegboard performance. Noteworthy, the Pegboard test was the only of the tests analyzed here which was sensitive to depressive symptoms (reduced performance with more depressive symptoms) of the subjects, and it was also the only test which indicated performance differences between never and former smokers. Thus, when it comes to sensitivity for the age, sex, and other variables describing community-dwelling, middle-aged to elderly adults, there appeared to be no advantage of the Q-Motor tests over the Purdue Pegboard test. However, it is quite possible that the Q-Motor tests would demonstrate advantages with respect to clinical samples, for instance and most likely in case of HD or PD. The Pegboard test is clearly the most complex of the tasks analyzed here, and thus it is not too surprising that this task proved sensitive to the largest number of variables analyzed here. However, in clinical samples, the task complexity may present challenges and may limit the applicability and proper execution depending on the stage or severity of disease. Moreover, complex tasks are more likely to be affected by learning and placebo effects (26). Q-Motor, in contrast, repeatedly exhibited lack of placebo effects [e.g., (4, 28)]. Furthermore, the sensitivity of Q-Motor assessments allowed detection of progression of motor changes even in pre-manifest HD gene carriers both cross-sectionally (56) and longitudinally (26). The high level of sensitivity and the rater-independence are important features of the Q-Motor tests, which may open unique opportunities for precise phenotyping of samples in future studies.

### Limitations

The data analyzed here originate from an observational study, and the current analyses were cross-sectional. Thus, the effects of age and sex on the Q-Motor outcomes found here may or may not reflect causal effects. Moreover, the current sample was “selected” to a certain degree, because the subjects had volunteered for participation in the BiDirect Study in the first place, because only a subset of the participants originally enrolled had returned to the second BiDirect visit, and because subjects with certain conditions had been excluded from the current analyses. As a consequence, the current sample and results may not be representative for the general population of a mid-sized, north-western European city. Further, it cannot be excluded that the effects found here may be biased due to residual confounding. Furthermore, the sample included mainly right-handers, and handedness determination was limited to asking subjects which hand they preferred. Thus, the findings should carefully be compared to studies employing different (possibly more sophisticated) hand preference determination. Moreover, average education of the subjects was rather high and bi-modally distributed. Finally, formal performance validity testing was not employed, allowing the possibility that the subjects may not have performed with full motivation or effort.

## Conclusions

The present results show specific effects of age and sex on Q-Motor speeded finger tapping and grasping and lifting performance. In addition, besides effects of education, there also were specific effects of smoking status and alcohol consumption. The latter were restricted to grasping and lifting and potentially due to acute substance deprivation. These are important findings, which imply that these variables may confound associations with Q-Motor outcomes potentially found in future (e.g., clinical) studies, and that careful reasoning about potential effects of these variables during the design of Q-Motor studies is vital.

It is also important to emphasize that the present results fill another knowledge gap: this study is the first to provide normative Q-Motor data from a large population-based sample. The normative data presented here can serve to inform the analysis and interpretation of Q-Motor data from future population-based or clinical studies.

Overall, the present results are in favor of the feasibility and validity of Q-Motor speeded finger tapping and grasping and lifting for large observational studies, albeit there were indications that care should be taken with regard to examiner training.

Comparisons of the effects on Q-Motor performance with effects on Purdue Pegboard performance in the setting of a large population-based observational study indicated that the Q-Motor tests may generate additional value in particular with regard to clinical conditions such as Huntington's or Parkinson's disease, where low task-complexity and lack of placebo effects are important preconditions.

## Data availability statement

The data of the BiDirect Study, including the data used and/or analysed during the current study, are available via the last author (KB) upon reasonable request.

## Ethics statement

The studies involving human participants were reviewed and approved by Ethik-Kommission der Ärztekammer Westfalen-Lippe und der Westfälischen Wilhelms-Universität Münster, Münster, Germany. The patients/participants provided their written informed consent to participate in this study.

## Author contributions

HT and KB conceived of the present study. HT performed the statistical analyses, interpreted the results, and drafted the manuscript. RR provided the Q-Motor apparatus. RS performed the blinded Q-Motor data pre-analyses. HT, KB, RS, and RR participated in the interpretation of the findings, contributed to the writing of the manuscript, and approved the final version of the manuscript. All authors contributed to the article and approved the submitted version.

## Funding

The BiDirect Study was funded by the German Federal Ministry of Education and Research (Grant Nos: 01ER0816 and 01ER1506 to KB). We acknowledge support from the Open Access Publication Fund of the University of Münster.

## Conflict of interest

Author RS is an employee of the George Huntington Institute (GHI), a private research institute focused on clinical and preclinical research in Huntington's disease, and QuantiMedis, a clinical research organization providing Q-Motor (quantitative motor) services in clinical trials and research. Author RR is founding director and owner of the George-Huntington-Institute, a private research institute focused on clinical and preclinical research in Huntington's disease, and QuantiMedis, a clinical research organization providing Q-Motor (quantitative motor) services in clinical trials and research. He also serves as a member of the Task Force on Technology of the International Parkinson and Movement Disorder Society (IPMDS). He has provided consulting services, advisory board functions, clinical trial services, quantitative motor analyses, and/or lectures for Actelion, Alnylam, Amarin, Annexion, AOP Orphan Pharmaceuticals, Cure Huntington Disease Initiative Foundation (CHDI), Desitin, Hoffmann-La Roche, IONIS, Ipsen, Lundbeck, MEDA Pharma, Medivation, Mitoconix, Neurosearch, Novartis, Omeros, Pfizer, Prana Biotechnology, Prilenia, PTC Therapeutics, Raptor, Siena Biotech, Temmler Pharma, Teva, uniQure, Vaccinex, VectorY, Voyager, and Wave Life Sciences. The remaining authors declare that the research was conducted in the absence of any commercial or financial relationships that could be construed as a potential conflict of interest.

## Publisher's note

All claims expressed in this article are solely those of the authors and do not necessarily represent those of their affiliated organizations, or those of the publisher, the editors and the reviewers. Any product that may be evaluated in this article, or claim that may be made by its manufacturer, is not guaranteed or endorsed by the publisher.
