# Supplementary material for: Effects of age and sex on outcomes of the Q-Motor speeded finger tapping and grasping and lifting tests-findings from the population-based BiDirect Study
Source: Front Neurol. 2022 Sep 30;13:965031. doi: 10.3389/fneur.2022.965031 (PMC9561931; doi:10.3389/fneur.2022.965031)
Supplement: Supplementary file 1 [file Image_1.pdf]

Supplementary Material to Teismann et al. 2022

Supplementary Material to Teismann et al. 2022

1 Supplementary Figures

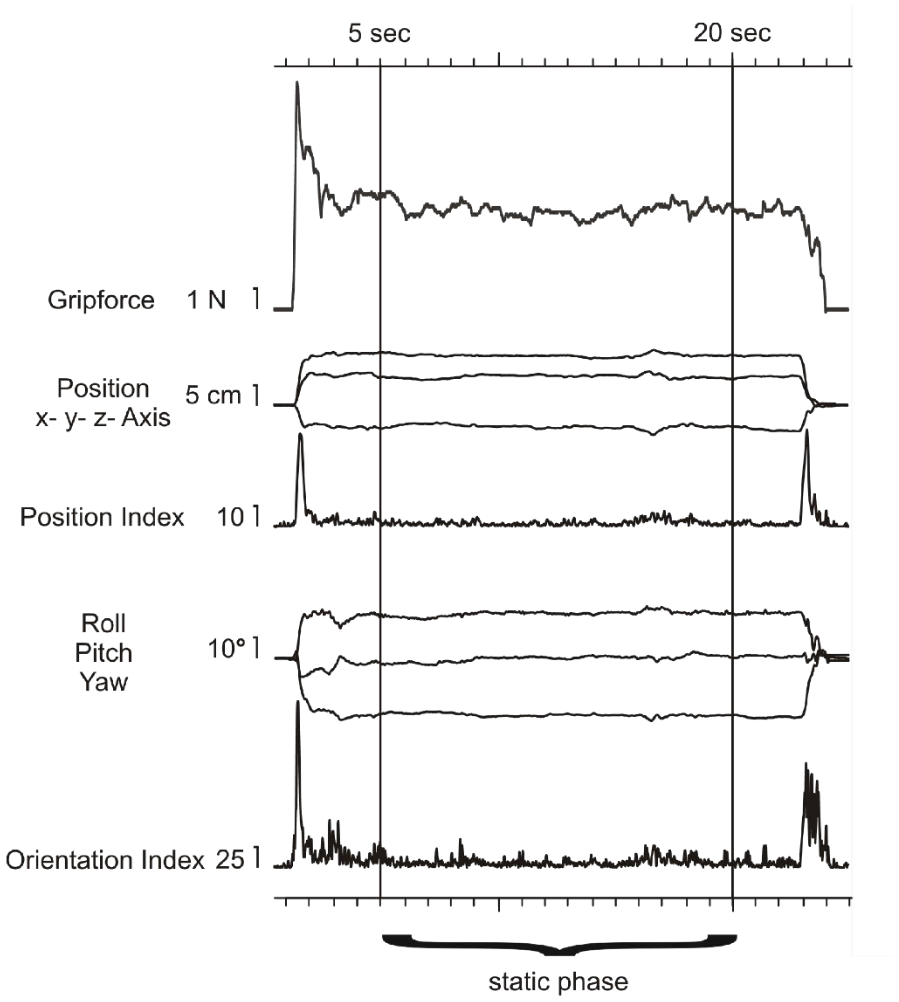

Supplementary Figure 1. A typical example of a raw signal recorded during a grasping and lifting trial, together with depictions of outcome measures (position/ orientation index) which can be derived from the raw signal. Figure modified from "Reilmann R (2012) Huntington's disease: towards disease modification – gaps and bridges, facts and opinions. Basal Ganglia 2: 241–248"

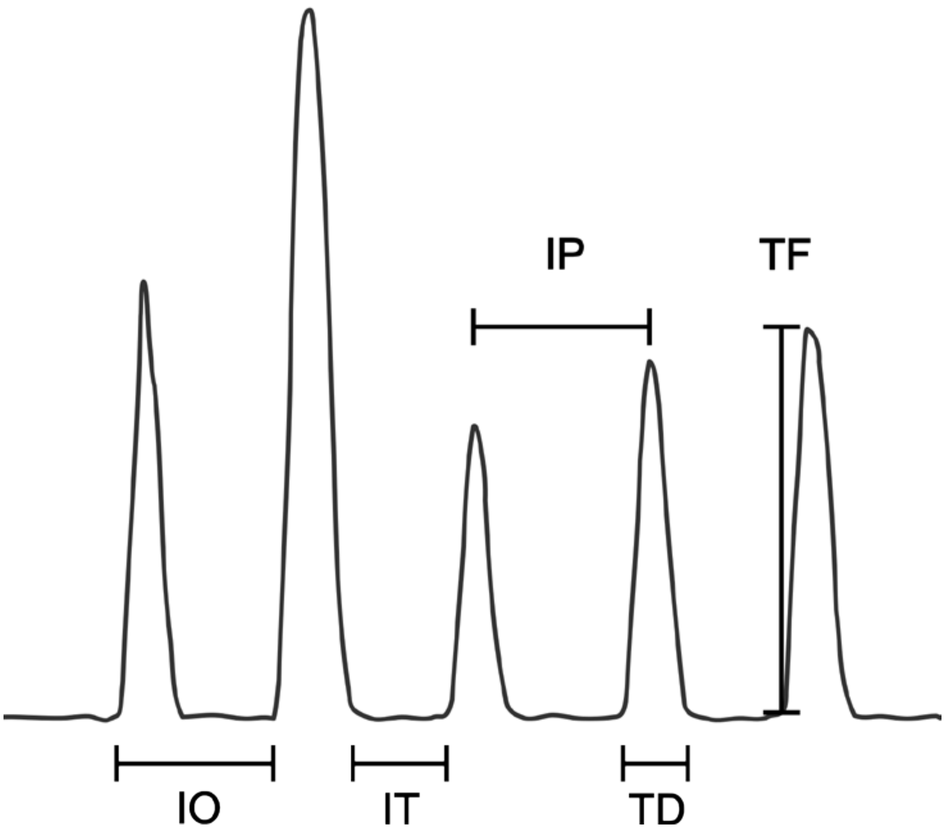

Supplementary Figure 2. A typical example of a raw signal excerpt recorded during a speeded finger tapping trial, together with depictions of outcome measures (IO: inter-onset interval; IT: inter-trial interval; IP: inter-peak interval; TD: trial duration; TF: tap force) which can be derived from the raw signal. Picture by George-Huntington-Institute (GHI) / CC BY-ND 4.0.

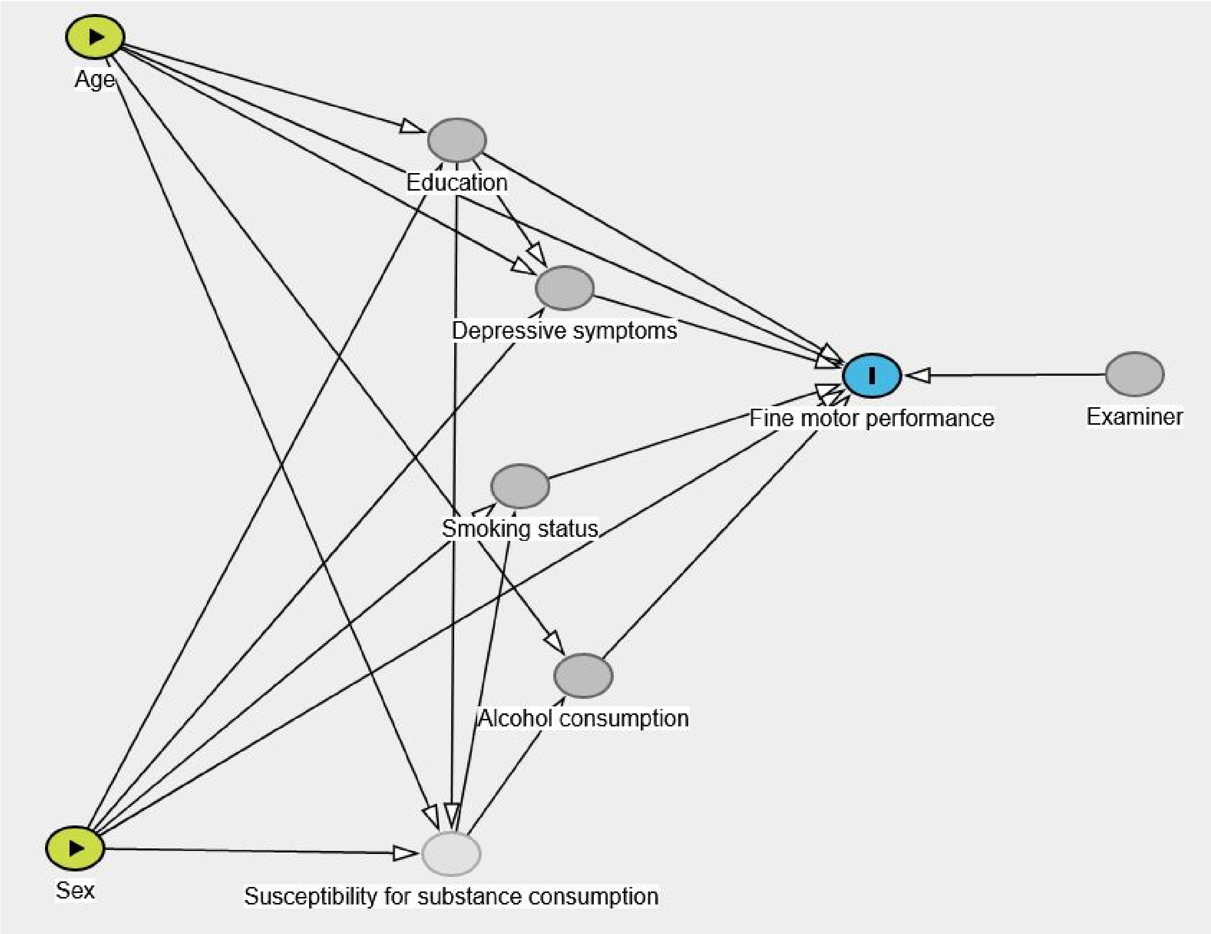

Supplementary Figure 3. Directed acyclic graph (DAG; cf. Textor et al., 2016) representing presumed causal relationships among the explanatory variables of interest age and sex (green; "exposure"), fine motor performance (blue; "outcome"), and other observed (dark grey) or unobserved (light grey) explanatory variables (possible "mediators" or "confounders", respectively). Given this DAG, Model 2 (cf. section "2.6 Analysis details") approximates direct effects of age and sex on fine motor performance. Edges (or arrows, respectively) depict causal paths.

Reference:

Johannes Textor, Benito van der Zander, Mark S. Gilthorpe, Maciej Liskiewicz, and George TH Ellison. Robust causal inference using directed acyclicgraphs: the R package 'dagitty'. International Journal of Epidemiology, 45(6):1887–1894, December 2016.

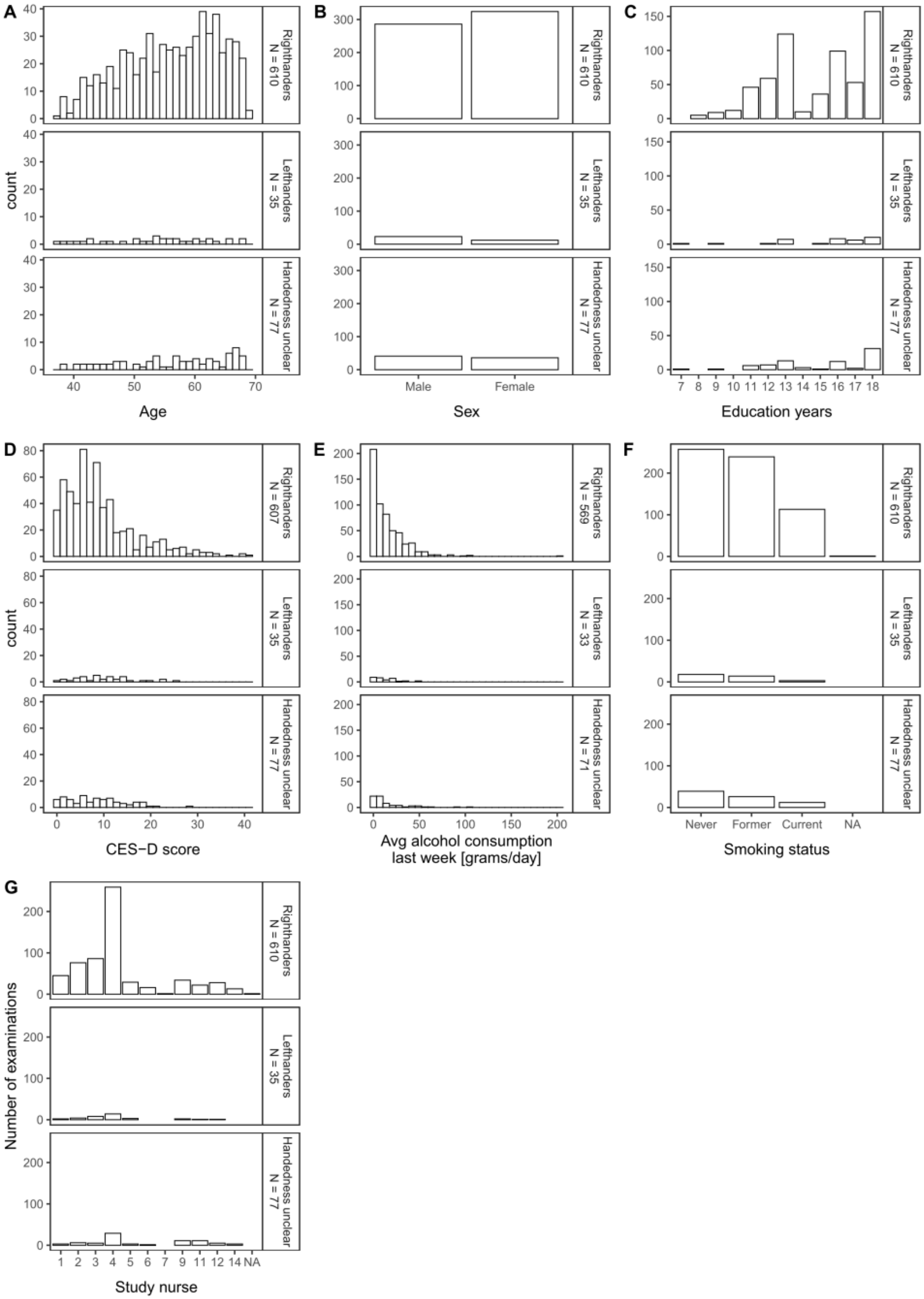

Supplementary Figure 4. Univariate distributions of explanatory variables, stratified by handedness.

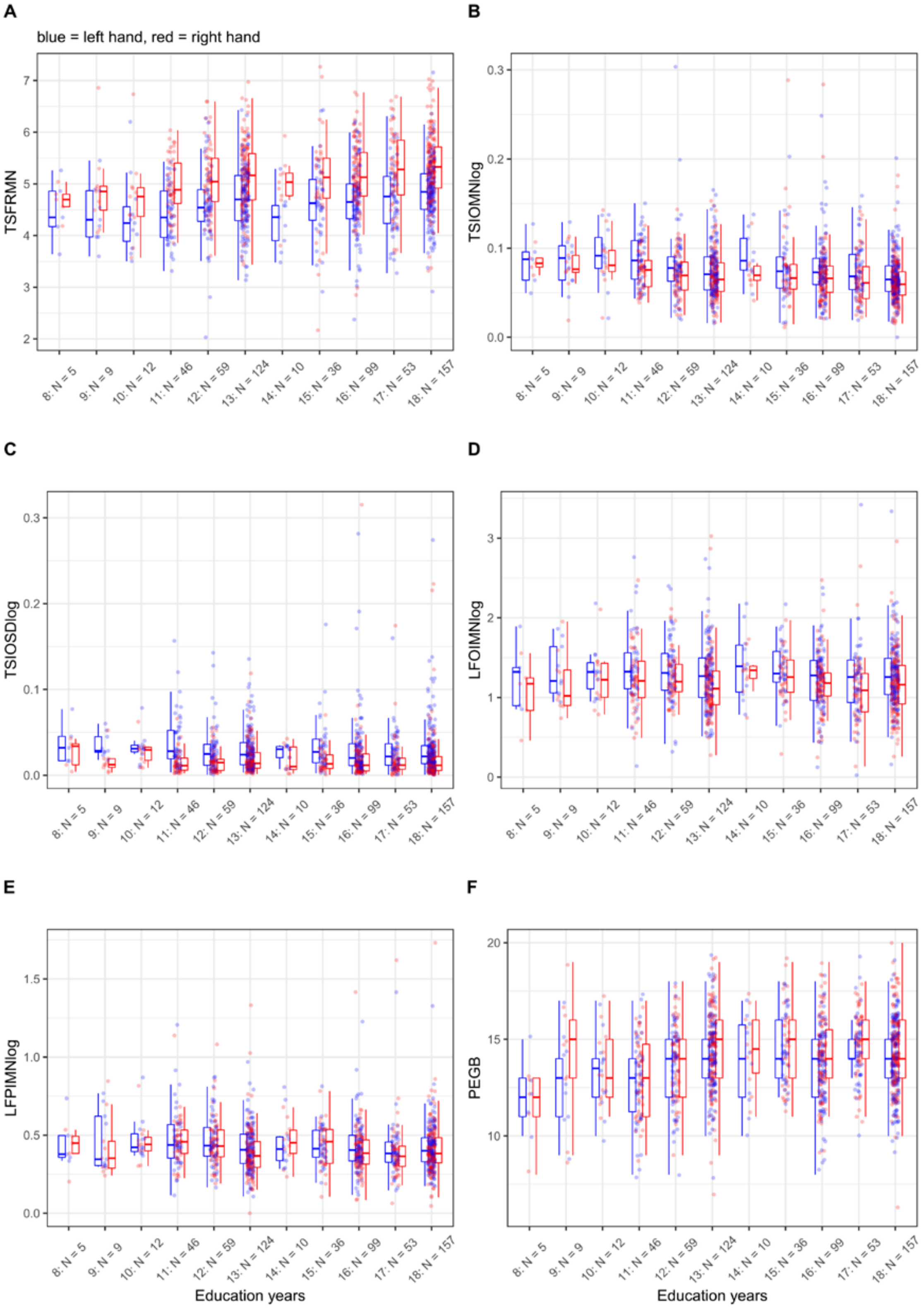

Supplementary Figure 5. Distributions of motor outcomes variables stratified by education years and handedness.

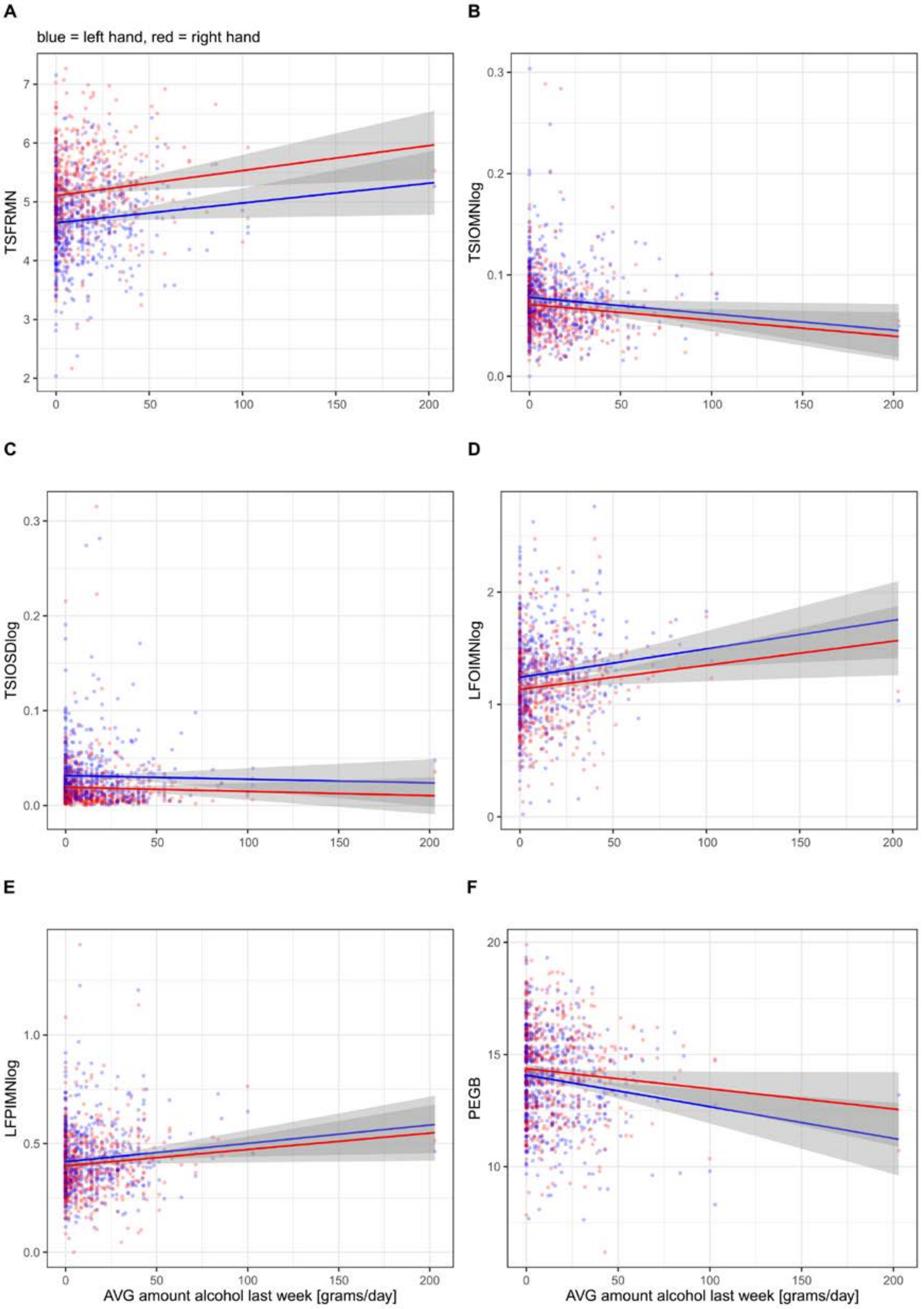

Supplementary Figure 6. Associations of motor outcomes with the average amount of alcohol consumed during the last seven days stratified by handedness.

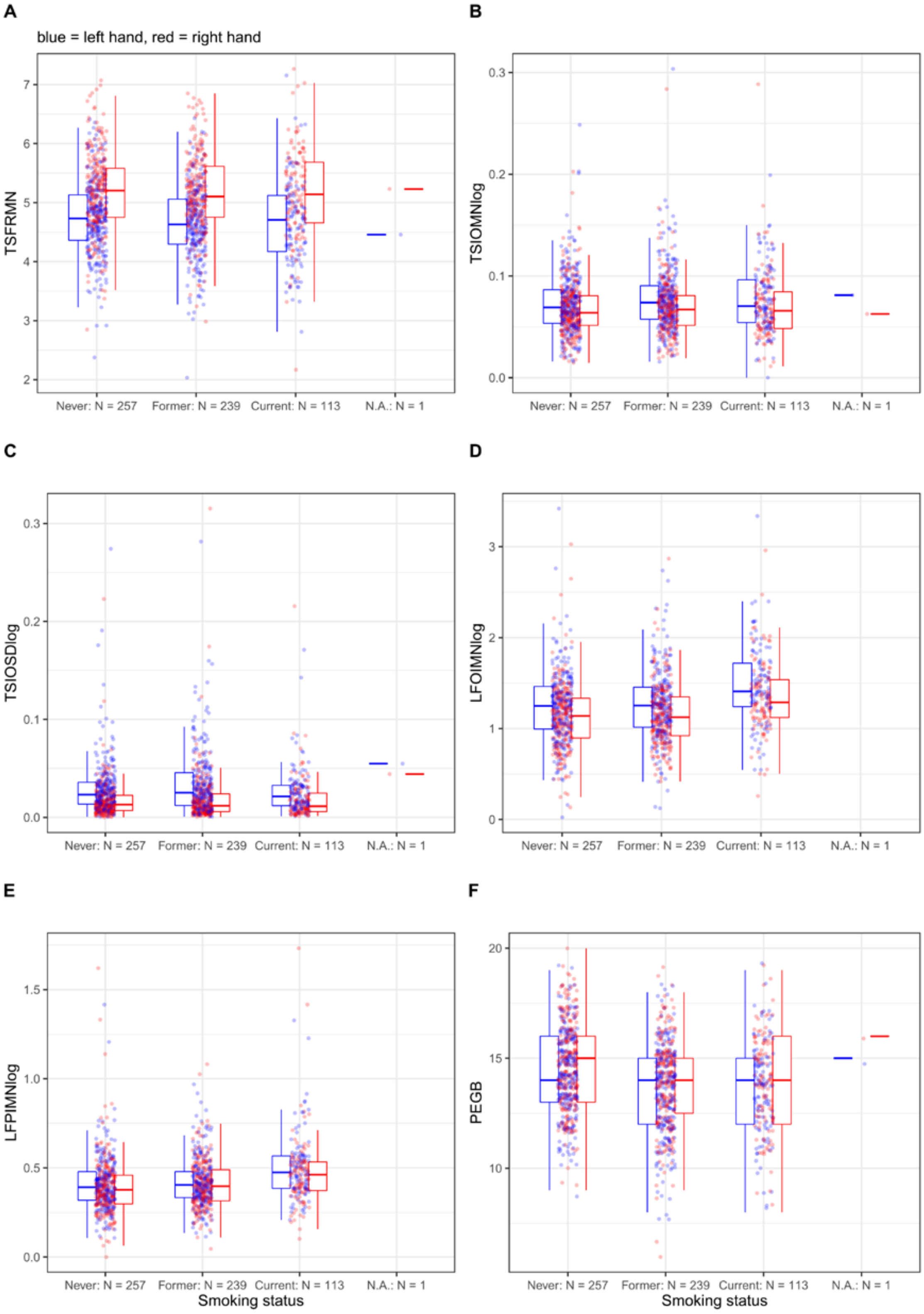

Supplementary Figure 7. Distributions of motor outcomes variables stratified by smoking status and handedness.

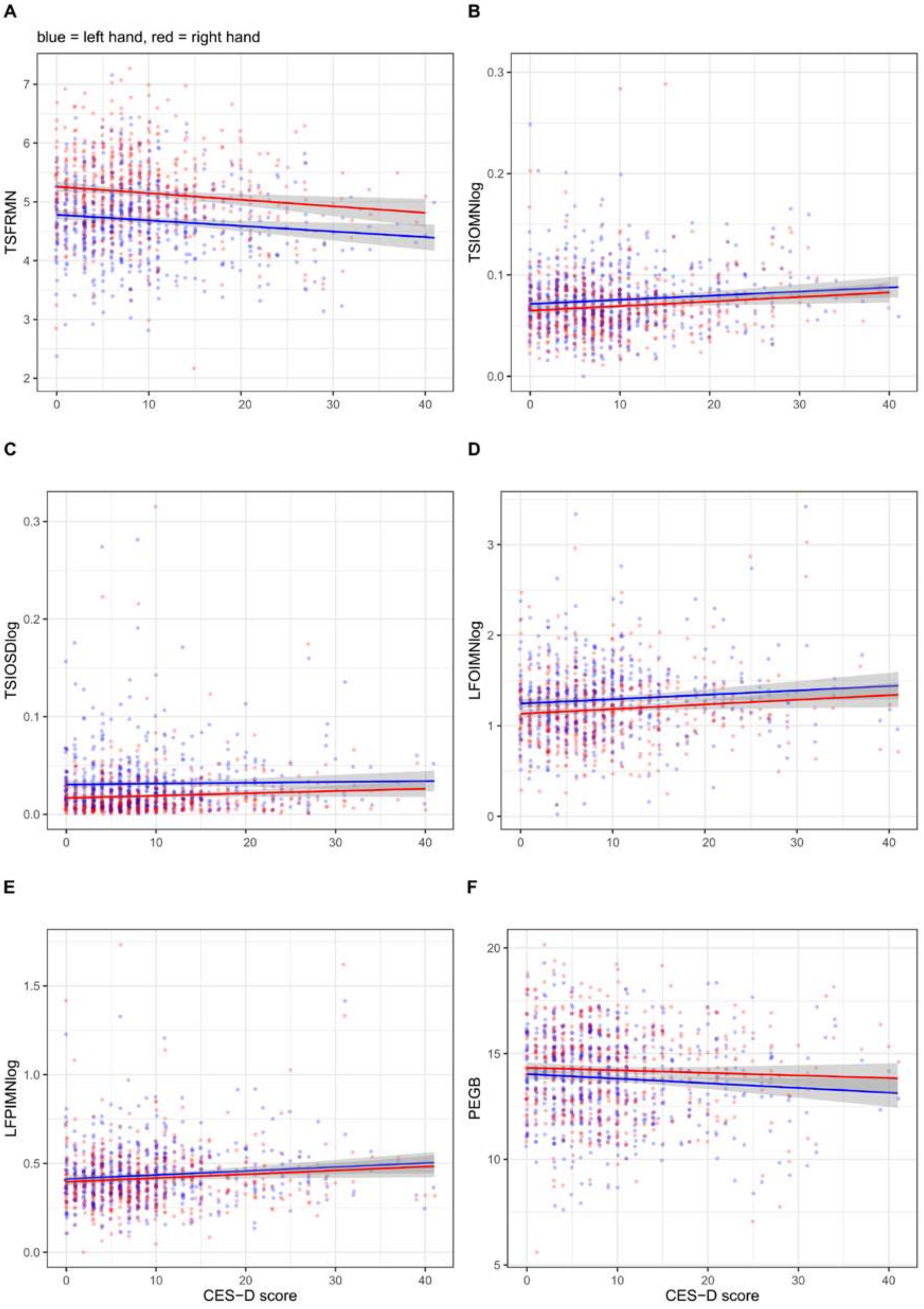

Supplementary Figure 8. Associations of motor outcomes with severity of depressive symptoms during the past week stratified by handedness.

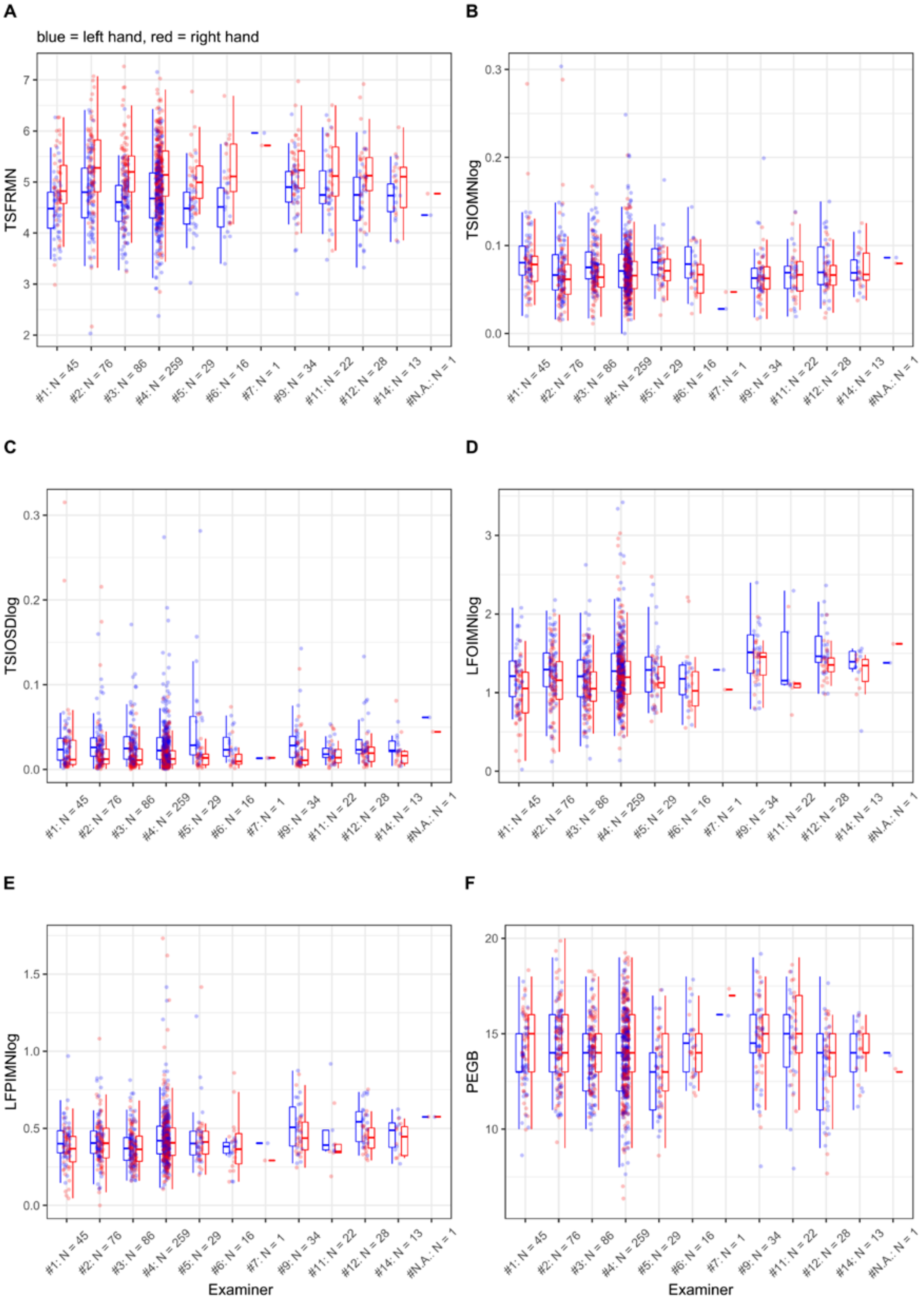

Supplementary Figure 9. Distributions of motor outcomes variables stratified by examiner and handedness.

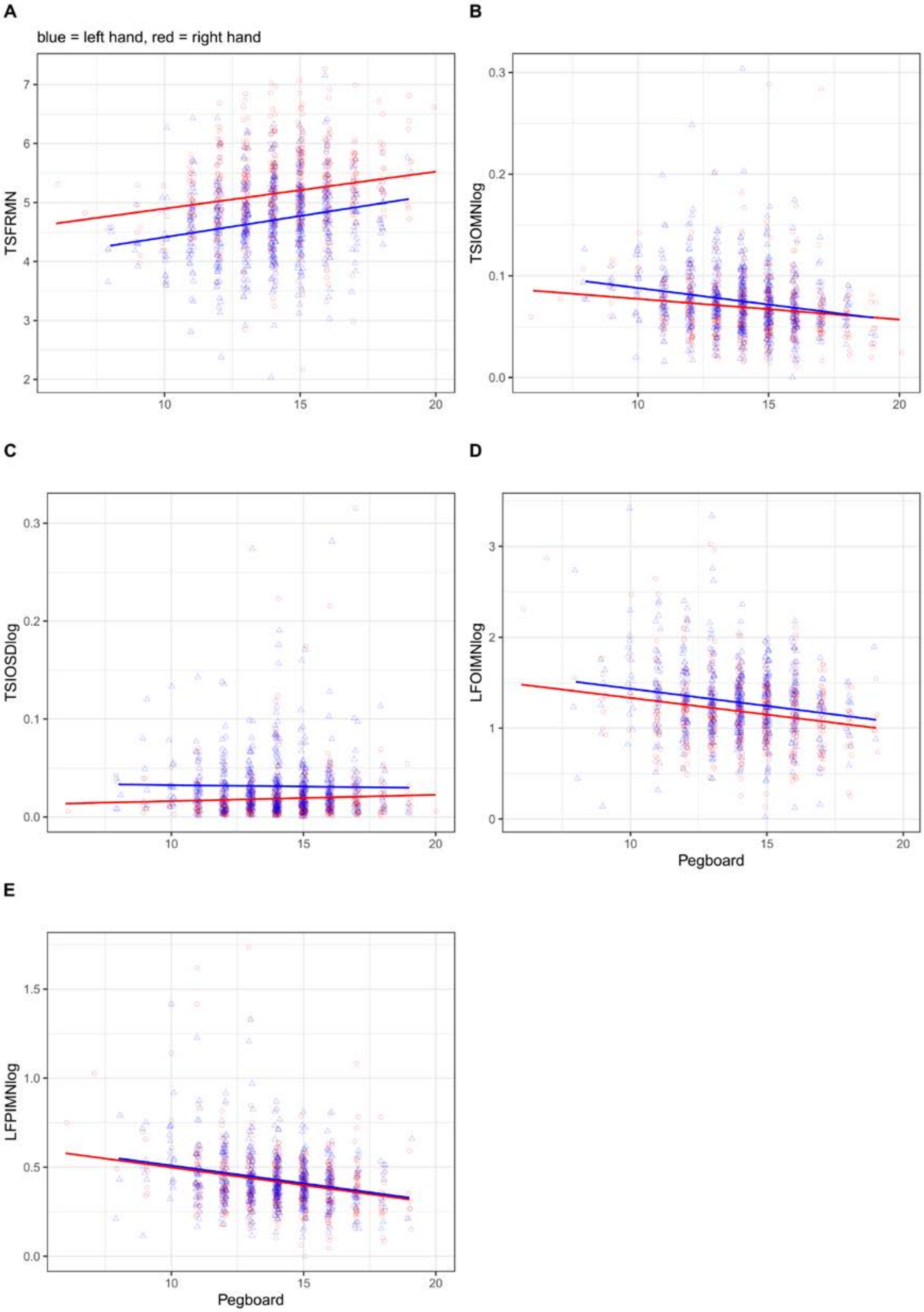

Supplementary Figure 10. Associations of Q-Motor outcomes with the Pegboard outcome, stratified by handedness.
